# A microscale soft lithium-ion battery for tissue stimulation

**DOI:** 10.1038/s44286-024-00136-z

**Published:** 2024-10-25

**Authors:** Yujia Zhang, Tianyi Sun, Xingyun Yang, Linna Zhou, Cheryl M. J. Tan, Ming Lei, Hagan Bayley

**Affiliations:** 1 Department of Chemistry, University of Oxford, Oxford, UK; 2 Institute of Electrical and Microengineering, École Polytechnique Fédérale de Lausanne, Lausanne, Switzerland; 3 Department of Pharmacology, University of Oxford, Oxford, UK; 4 Ludwig Institute for Cancer Research, Nuffield Department of Medicine, University of Oxford, Oxford, UK; 5 Division of Cardiovascular Medicine, Radcliffe Department of Medicine, University of Oxford, Oxford, UK

**Keywords:** Biomedical engineering, Batteries, Gels and hydrogels

## Abstract

Advances in the development of tiny devices with sizes below a few cubic millimeters require a corresponding decrease in the volume of driving power sources. To be minimally invasive, prospective power sources in biomedical devices must be fabricated from soft materials. Previous endeavors with droplet-based devices have produced promising miniature power sources; however, a droplet-based rechargeable battery has remained out of reach. Here we report a microscale soft flexible lithium-ion droplet battery (LiDB) based on the lipid-supported assembly of droplets constructed from a biocompatible silk hydrogel. Capabilities such as triggerable activation, biocompatibility and biodegradability and high capacity are demonstrated. We have used the LiDB to power the electrophoretic translocation of charged molecules between synthetic cells and to mediate the defibrillation and pacing of ex vivo mouse hearts. By the inclusion of magnetic particles to enable propulsion, the LiDB can function as a mobile energy courier. Our tiny versatile battery will thereby enable a variety of biomedical applications.

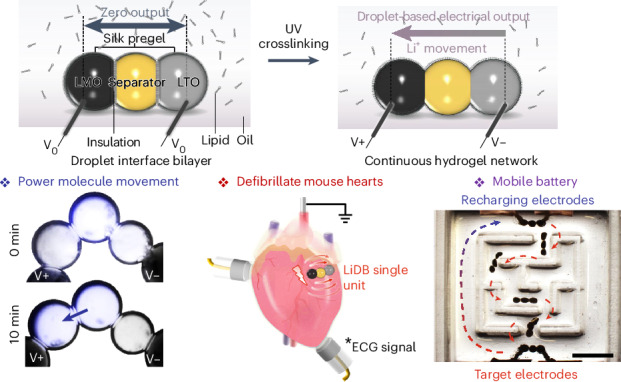

## Abstract (web summary)

The development of tiny, soft and biocompatible batteries to power minimally invasive biomedical devices is of critical importance. Here the authors present a microscale soft rechargeable lithium-ion battery based on the lipid-supported assembly of silk hydrogel droplets that enables a variety of biomedical applications.

## Main

The miniaturization of electronic devices is a burgeoning area of research^1–3^. Therefore, the development of tiny batteries to power these devices is of critical importance, and techniques such as three-dimensional (3D) printing^4–6^ and micro-origami assembly^7^ are beginning to have an impact. For minimally invasive applications in biomedicine, batteries are also preferred to be soft, biocompatible and biodegradable, with additional functionality and responsiveness, such as triggerable activation and remote-controlled mobility^8^. However, at present, such a multifunctional microscale soft battery is not available. Although hydrogel-based lithium-ion (Li-ion) batteries demonstrate some of these features^9–12^, none currently exhibits microscale fabrication of the battery architecture, in terms of self-assembled integration of hydrogel-based cathode, separator and anode at the submillimeter level. Manual assembly of precrosslinked compartments^11^ or multistep deposition and crosslinking^4^ is necessary to avoid the mixing of materials from different compartments at the pregel (liquid) state or during the gelation process. This limitation not only makes it difficult to shrink hydrogel-based functional architectures but also hinders the implementation of high-density energy storage.

Toward that end, Zhang et al. have reported a miniaturized ionic power source by depositing lipid-supported networks of nanoliter hydrogel droplets^13^. The power source mimics the electrical eel by using internal ion gradients to generate ionic current^14^, and can induce neuronal modulation. However, the ionic power source has several limitations that should be addressed. First, the stored salt gradient produces less power than conventional Li-ion batteries, and the device cannot be fully recharged. Second, activation of the power source relies on temperature-triggered gelation and oil for buffer exchange, which is a demanding requirement. Third, the functionality of the power source is limited to the generation of ionic output, leaving the full versatility of synthetic tissues unexploited^15–17^. Last, but not least, while the power source can modulate the activity of neural microtissues, organ-level stimulation necessitates a higher and more stable output performance in physiological environments^18^.

Here, we present a miniature, soft, rechargeable Li-ion droplet battery (LiDB) made by depositing self-assembling, nanoliter, lipid-supported, silk hydrogel droplets. The tiny hydrogel compartmentalization produces a superior energy density. The battery is switched on by ultraviolet (UV) light, which crosslinks the hydrogel and breaks the lipid barrier between droplets. The droplets are soft, biocompatible and biodegradable. The LiDBs can power charge molecule translocation between synthetic cells, defibrillate mouse hearts with ventricular arrhythmias and pace heart rhythms. Further, the LiDB can be translocated from one site to another magnetically.

## Results

### Design and performance of LiDBs

**Fig. 1:**
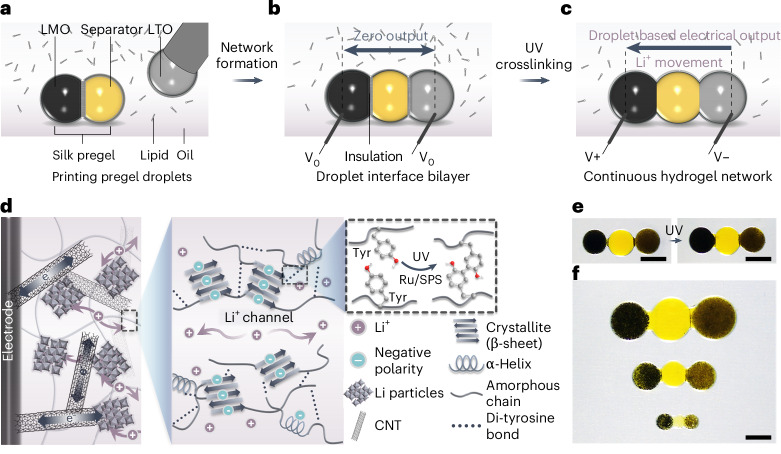
Design of the LiDB. **a**–**c**, Fabrication of a LiDB by consecutively depositing hydrogel droplets: silk pregel droplets were submerged in lipid-containing oil and acquired lipid monolayer coatings, which subsequently formed lipid bilayers when droplets were placed in contact (**a**); the lipid insulation prevented Li-ion flux between connected droplets (**b**); the battery was activated by UV crosslinking of the silk hydrogel, which ruptured the lipid bilayers to form a continuous hydrogel structure (**c**). Carbon electrodes were used to measure electrical output. V_0_, V+ and V− mark the zero output, outputs of the cathode and anode droplets respectively. **d**, The working mechanism of the LiDB. Electrons flow from electrodes to Li particles through conductive interconnecting CNTs. Charge neutralization then occurs by the intercalation or deintercalation of Li ions into or out of Li particles. Accordingly, Li ions flow throughout the negatively charged silk hydrogel, which is formed by dityrosine crosslinking (dashed box) mediated by a small number of tris(bipyridine)ruthenium(II) (Ru) ion, sodium persulfate (SPS) and low-intensity UV illumination (1 min). **e**, Bright-field images of a LiDB before and after UV-induced crosslinking. Droplet volumes, 30 nl. **f**, Bright-field images of LiDBs of different droplet volumes. From top to bottom: 250, 50 and 10 nl. Scale bars, 400 μm.

A single LiDB unit comprised three silk-hydrogel droplets that contained lithium manganese oxide (LiMn_2_O_4_, LMO) particles and carbon nanotubes (CNTs) in the cathode droplet, lithium titanate (Li_4_Ti_5_O_12_, LTO) particles and CNT in the anode droplet, and a central droplet containing lithium chloride (LiCl) as a separator (Fig. 1a; detailed material compositions are included in Methods). The cathode, separator and anode droplets were deposited in a lipid-containing oil by using a microinjector. The droplets were initially surrounded by monolayers of lipid, which formed droplet interface bilayers (DIBs) within seconds upon contact with one another, thereby creating a stabilized, support-free structure (Fig. 1b)^19,20^. The lipid-supported droplet assembly and silk fibroin together played critical roles in a LiDB. First, the silk solution readily mixes with various active components in the pregel state. After droplet deposition, the DIBs prevent material diffusion between different droplets, acting as an insulative physical barrier^21^. This approach differs critically from previous fabrication methods of hydrogel batteries, in which components were first separately cast and crosslinked, and then manually assembled to form the final battery^9–12^. Premature assembly would result in the mixing of active materials, which would produce short circuits and, hence, battery deactivation. The consequently cumbersome and time-consuming fabrication process limits the minimum size of a soft battery. In contrast, in our approach, the cathode, separator and anode droplets self-assemble into a tiny LiDB within seconds. The DIBs allow the construction of soft and free-standing hydrogel units with distinct compartments at microscale. Our fabrication device can print droplets as small as 0.5 nl, which are ~100 μm in diameter—a more than tenfold decrease compared with previous work^9–11^. Second, photochemical dityrosine crosslinking of the silk hydrogel by UV irradiation for 1 min (Supplementary Fig. 1) ruptured the DIBs (Supplementary Fig. 2) and established a pathway that conducts Li ions (Fig. 1c), thereby activating the LiDB (Fig. 1d,e). Third, after the formation of a hydrogel structure, silk as a static component had a plethora of favorable properties that provided mechanical stability and hosted the energy-generating machinery (Fig. 1d). The elastic behavior of the silk hydrogel ensured the robust performance of LiDBs^22^, including flexibility and compressibility with a modulus of ~10 kPa at 80% compression (Supplementary Fig. 3), and stable support of embedded Li particles and CNT, creating high electrical conductivity between the two electrodes (~41.3 mS cm^−1^; Supplementary Fig. 4a,b). The crosslinked silk fibroin enhanced the cation selectivity through its negatively charged amino acids, which showed an average zeta potential of −20.2 mV (Supplementary Fig. 4c) that facilitated Li-ion conduction^23^. Furthermore, the silk hydrogel is biocompatible^24–26^, biodegradable^27,28^ and strongly adhesive toward tissues^29^, properties that are essential for biological use. Therefore, given the benefits of using the lipid-supported assembly of silk fibroin-containing droplets (Fig. 1f), we have developed a new fabrication methodology to construct microscale soft batteries composed of biocompatible materials, by contrast with conventional bulky and rigid Li-ion batteries. Device production can be automated readily by using microfluidics and 3D droplet printers^19^.

**Fig. 2:**
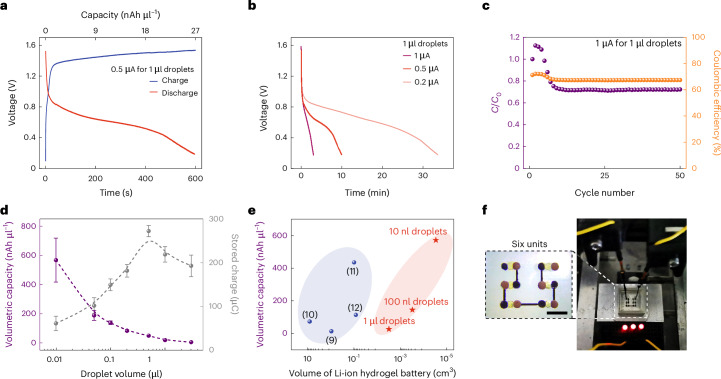
Electrochemical characteristics of LiDBs. **a**, Galvanostatic charge–discharge curves of LiDB at a current of 0.5 μA. **b**, Discharge curves of LiDB at different currents. **c**, Cyclic performance of LiDB at a current of 1 μA. *C*_0_ and *C* correspond to the capacities before and after the cycle. **d**, Calculated volumetric capacities and released charge of LiDBs with various droplet volumes. **e**, A comparison of volumetric capacities of LiDBs (red) and reported flexible Li-ion hydrogel batteries (blue)^9–12^. **f**, Six LiDB units were connected in series by underneath screen-printed carbon electrodes to light up three red light-emitting diodes. One microliter (**a**–**c**) and 0.5 μl (**f**) droplets were used. Scale bar, 2.4 mm (**f**). Data in **d** and **e** are presented as mean values ± standard deviations (s.d.) of *n* = 5 replicates. Source data

Cyclic voltammograms of cathode and anode hydrogel electrodes were explored with a three-electrode system, which revealed that the silk hydrogel was electrochemically stable within the working potential range and the redox reactions of both electrodes could be reversibly operated (Supplementary Fig. 5). A mass loading of 10% w/v Li particles in both hydrogel electrodes prevented particle aggregation (scanning electron microscope images in Supplementary Fig. 6 and volumetric capacities with various mass loadings in Supplementary Fig. 7). Galvanostatic charge–discharge curves exhibited an average discharge voltage plateau of 0.65 V (Fig. 2a). A promising output performance of the LiDB was realized, with volumetric capacities of 17, 27 and 46 nAh μl^−1^ at 1, 0.5 and 0.2 μA for 1 μl droplets (Fig. 2b). Moreover, the LiDB performed with high stability with an over 72% retention of output capacity after 50 cycles at 1 μA (Fig. 2c) and restored its output capacity after applying a lower charge–discharge current of 0.5 μA (Supplementary Fig. 8). The robust hydrogel compositions ensured a stable storage time of more than 48 h (Supplementary Fig. 9). Further, temperature changes during charging and discharging were negligible (Supplementary Fig. 10).

Miniaturization of LiDBs was facilitated by the lipid-supported droplet self-assembly. Decreasing the volume of the droplets increased the surface-to-volume ratio and thus provided more reaction sites per unit of volume during battery charging and discharging (Supplementary Note 1). Therefore, the miniaturization facilitated the redox reactions of hydrogel electrodes and enhanced the coulombic efficiency and volumetric capacity^3,30^. By shrinking the droplet volume from 3 μl to 10 nl, which resulted in a unit length of ~600 μm, the released charge of LiDBs was first observed to increase as the volume decreased from 3 μl to 0.5 μl, due to the enhanced coulombic efficiency, but then decreased with further volume reductions as a result of the reduction in the number of contained Li particles (Fig. 2d). During the 99.7% volume shrinkage, we observed a concomitant increase in the volumetric capacity, leading to a level of ~570 nAh μl^−1^, which is unprecedented for all-hydrogel Li-ion batteries; further, our LiDB is more than 10^3^-fold smaller than those in previous work (Fig. 2e and Supplementary Table 1)^9–12^. We also increased the output voltage of LiDBs by connecting multiple units in series in a custom-made well (Fig. 2f). Open-circuit voltage increased with the number of LiDBs in series, reaching ~3.3 V for six connected units (Supplementary Fig. 11), which powered three light-emitting diodes and other electronic devices, for example, a liquid-crystal display timer (Supplementary Fig. 12).

### Tetherless activation of charged molecule translocation

**Fig. 3:**
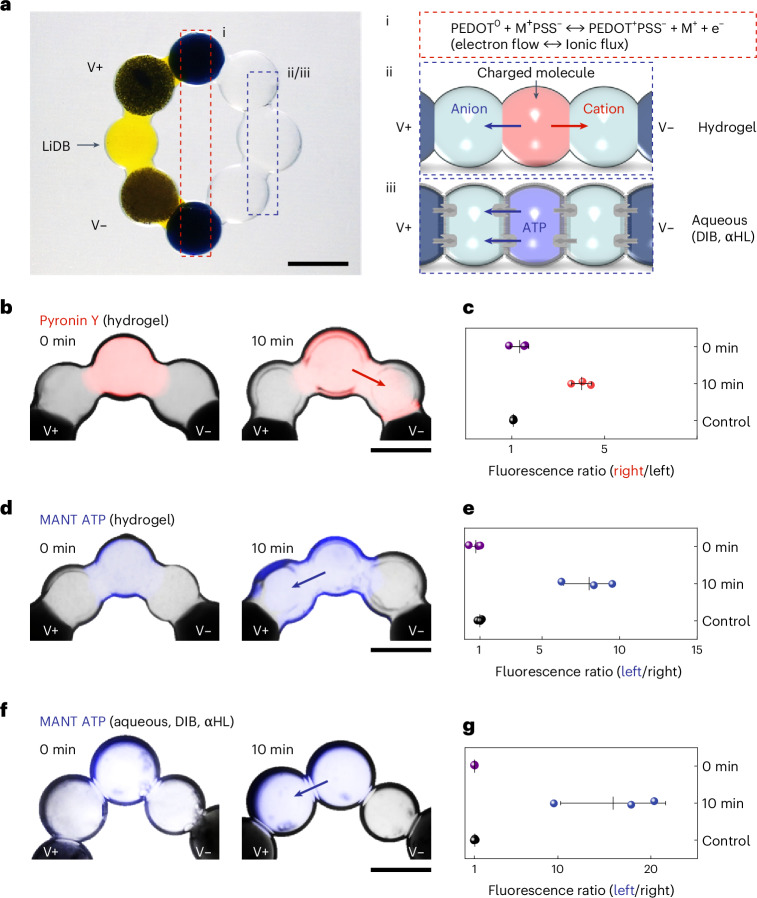
Charged molecule translocation powered by LiDBs. **a**, A bright-field image of a LiDB unit tetherlessly linked with two converting droplets (i, middle red box) and three hydrogel droplets (ii) or aqueous synthetic cells (iii, right blue box). The converting hydrogel droplets contained 1.1% w/v PEDOT:PSS that transformed the electron flow from a LiDB into ion flux (i). M^+^ represents the cation in the electrolyte. The pore-forming protein αHL was used to achieve signal (charged molecule) transmission through DIBs (iii). Fluorescent charged molecules were cationic pyronin Y (10 μM, red) and anionic MANT-dATP (100 μM, blue). V+ and V− mark the outputs of cathode and anode droplets. **b**, Bright-field and fluorescence microscopy overlays (background removed) of three hydrogel droplets at the start and after 10 min of the LiDB actuation. Initially, only the central cell contained pyronin Y. **c**, Fluorescence ratios indicating that pyronin Y moved toward the anode of the LiDB (the right droplet). **d**,**f**, Overlays (background removed) of hydrogel droplets (**d**) and aqueous cells (**f**) containing MANT-dATP at the start and after 10 min of the LiDB actuation. **e**,**g**, Fluorescence ratios indicating that MANT-dATP moved toward the cathode of the LiDB (the left droplet) without (**e**) and with DIBs (**g**). Droplet volumes, 0.3 µl. Scale bars, 800 μm. Data in **c**, **e** and **g** are presented as mean values ± s.d. of *n* = 3 replicates. Source data

Synthetic tissues are networks of patterned communicating aqueous compartments, with potential for biomedical applications, including the release of therapeutic agents and the repair of damaged organs^31^. To electrically drive the communication between synthetic cells, previous work employing droplet networks demonstrated tetherless powering by light-driven proton pumps^32^. Nevertheless, such light-powered charged molecule translocation was slow, taking over 15 h to transmit over ~10 mm distance owing to the low generated current (~20 pA). The high-output, aqueous, microscale and soft features make the droplet-based LiDB a prospective approach to interface with synthetic cells, providing a direct and tetherless input electric field as well as offering improved stability and pliability. A pair of electron-to-ion converting droplets were devised to convert the electron current into ion flux (Fig. 3a). We used poly(2,3-dihydrothieno-1,4-dioxin)-poly(styrenesulfonate) (PEDOT:PSS) as an effective redox-active material for the conversion (Supplementary Fig. 13; see Methods for detailed compositions)^33,34^. The LiDB with converting droplets connected to its cathode and anode can be attached to other droplets through its termini, generating ion flux that would drive electrophoresis in the connected droplets^32^, drawing cations to the anode and anions to the cathode.

To demonstrate charged molecule translocation, the fluorescent cationic pyronin Y and anionic 3′-*O*-(*N*-methylanthraniloyl) adenosine 5′-triphosphate (MANT-dATP) were chosen. Both hydrogel droplets without DIBs and aqueous droplets with DIBs, but connected by the pore-forming protein α-hemolysin (αHL), thereby acting as synthetic cells^19,32^, were tested to demonstrate signal transmission in the absence of electrical tethers (that is, without metal electrodes). A droplet incorporating a fluorescent dye was put at the central position sandwiched between two droplets containing no dye, and together attached to the LiDB with the converting droplets. Subsequent dye movement was dictated by the interplay between spontaneous diffusion and electrophoresis, and was monitored by the observation of fluorescence changes. After subjection to 10 min of LiDB power, the cationic pyronin Y moved to the anode side (Fig. 3b,c), whereas the anionic MANT-dATP moved to the cathode side (Fig. 3d,e). In the case of aqueous droplets seperated by the DIBs, charged molecules were still successfully propelled into the target cell through the αHL pores embedded within the DIBs (Fig. 3f,g). We quantified the signal transmission by calculating the fluorescence ratio of the target droplet over the nontarget droplet, for example, cathode droplet over anode droplet for MANT-dATP (Supplementary Fig. 14). The aqueous synthetic cells showed a higher fluorescence ratio than their hydrogel counterparts, which was probably produced by the inhibition of spontaneous molecule diffusion by the DIBs and, therefore, less molecular accumulation in the nontarget cell.

### Ex vivo murine heart defibrillation by LiDBs

Electrical defibrillation is achieved by applying a strong electric field to the fibrillating heart, which restores sinus rhythm. Examples include automated external defibrillators in public places for patients with out-of-hospital cardiac arrest and implantable cardiac defibrillators for long-term treatments. However, the high-energy electric shock far exceeds the pain threshold and has a well-documented negative impact on quality of life, including post-traumatic stress disorder and depression^35^. The need for more efficient, low-energy, biocompatible defibrillation has promoted the development of miniature pacemakers^36^, implantable energy transducers^37–39^ and wireless power sources^18^.

**Fig. 4:**
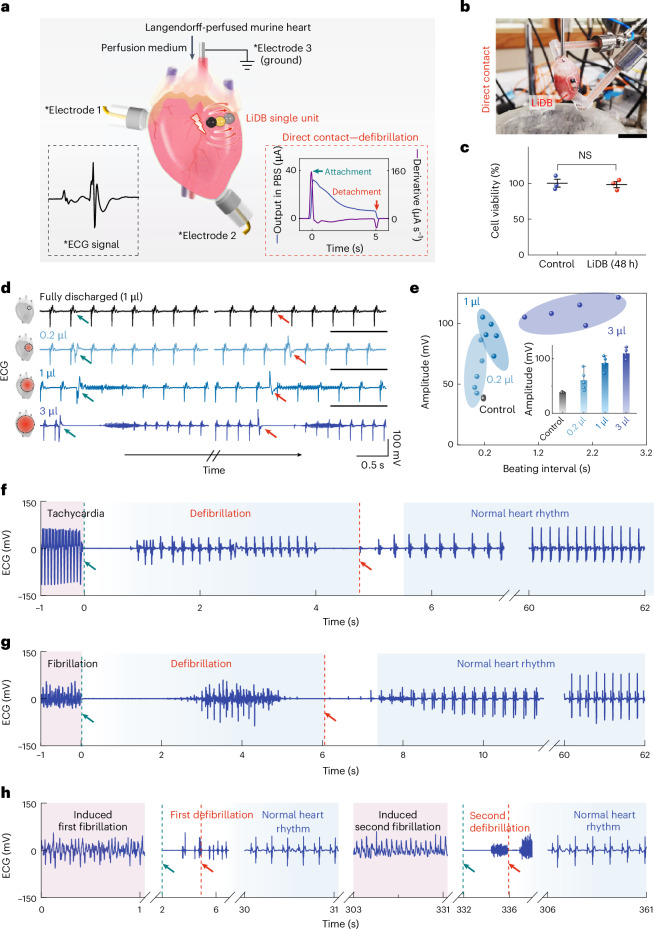
Ex vivo murine heart stimulation by LiDBs. **a**, Direct contact by a LiDB to produce electrical shock/defibrillation. Black box, an ECG signal was recorded with three electrodes to monitor the responses of the heart. Red box, the output current of the LiDB in phosphate-buffered saline (PBS), which flows through the heart during the contact process. The time derivative of the output current indicates that the maximum alterations in electrophysiological activity occur at the times when a LiDB is attached to a heart and detached from it. **b**, An image of the direct contact. Scale bar, 1 cm. **c**, Cell viability assay of fibroblasts after 48 h contact with fully charged LiDBs formed from 1 μl droplets (*n* = 3 replicates). NS, not significant; two-sided *t*-test. **d**, ECG traces of hearts contacted by LiDBs with droplet volumes of 0.2, 1 and 3 µl. The green arrows mark times of attachment, and the red arrows mark times of detachment. **e**, Shock amplitudes and beating intervals of hearts under stimulation by LiDBs with different droplet volumes (*n* = 5 independent mouse hearts). The beating interval was the time gap between the time of attachment and the next intrinsic heartbeat. Inset: comparison of mean shock amplitudes. The control group stands for fully discharged LiDBs (intrinsic heartbeats). **f**,**g**, ECG traces of heart defibrillation from ventricular tachycardia (**f**) and fibrillation (**g**). The heart was subjected to ouabain solution perfusion to induce the arrhythmia (Methods). The attachment (green arrow and dashed line) and detachment (red arrow and dashed line) of a LiDB were set as the start and end of a defibrillation process. The heart defibrillations were performed by LiDBs with 3 μl droplets. **h**, An ECG trace of a heart undergoing two sequential cycles of ouabain-induced fibrillation and LiDB-enabled defibrillation with 3 μl droplets. Data in **c** and **e** are mean values ± s.d. Source data

In this regard, our LiDBs can produce a direct current electrical discharge (shock) with miniaturized energy outputs and high modulation efficacy when in direct contact with Langendorff-perfused murine hearts as reflected in epicardial electrocardiograms (ECGs) (Fig. 4a,b). A LiDB was gently placed onto the surface of a heart, and the hydrogel adhered directly to the epicardium (Fig. 4b and Supplementary Video 1). Maximum effects occurred at the times of attachment and detachment, and were capable of mediating defibrillation. The electrical stimulation and the continuous contact of LiDBs showed no adverse influence on the heart tissue. Biocompatibility of LiDBs was confirmed by co-culturing them with mouse fibroblasts (Fig. 4c), human dermal fibroblasts or cardiomyocytes derived from human induced pluripotent stem (iPS) cells. Live/dead staining was performed after 48 h to examine the cell viability and proliferation (Supplementary Fig. 15a,b). After the LiDBs’ electrical discharge during co-culture, the cells reached a high density similar to the value of the control group and exhibited a typical spreading and intact filamentous morphology. Further results of cell viability, cytotoxicity and apoptosis assays conducted after 7 days of co-culture demonstrated the cytocompatibility of LiDBs (Supplementary Fig. 15c–e).

In conjunction with the LiDB stimulation, we conducted optogenetic light pacing to generate a regular heart rhythm (Supplementary Fig. 16), which eliminated ectopic heartbeats and thereby permitted an accurate manifestation of the heartbeats triggered by the LiDB stimulation (see Methods and Supplementary Fig. 17 for details of the optogenetic regulation)^40^. In contrast with a fully discharged LiDB, a charged LiDB imposed its electrical output on the heart and disrupted the intrinsic electrical activity of the heart muscle (Fig. 4d). The temporary change of electrical activity produced by the LiDB was able to restore normal heart rhythm in unsynchronized cardioversion (defibrillation) as demonstrated in the corresponding ECG traces. Increasing the volume of the droplets in the LiDB enlarged the contact area for stimulation, which increased the amplitude of the shock and temporarily suppressed the intrinsic heartbeats (Fig. 4e and Supplementary Fig. 18). We verified the involvement of an electrical shock by attaching a pair of electrodes onto the epicardium of hearts and applying direct current ranging from 15 to 45 µA (Supplementary Fig. 19). The stimulation by a LiDB comprising 1 µl droplets was equivalent to a ~30 µA direct current stimulation, showing a similar shock signal and subsequent heartbeat restoration in the ECG trace (Supplementary Figs. 20 and 21).

The electrical shock provided the opportunity to treat ventricular arrhythmias, for example, ventricular tachycardia and fibrillation, which are injurious in minutes if not treated promptly. The injection of ouabain solution (300 μl, 0.3 mg ml^−1^) led to ventricular tachycardia due to cytosolic calcium overload in heart cells, which gradually transformed into ventricular fibrillation (Supplementary Fig. 22). The attachment and detachment of a LiDB were set as the start and end of a defibrillation process. Applying the LiDB by direct contact enabled the defibrillation process within 5 s and converted ventricular arrhythmias to normal heart rhythms (Fig. 4f,g). After removing the LiDB, the heart recovered to sinus rhythm within 5 s without deterioration in heart function. The LiDBs can be applied to hearts repeatedly when multiple arrhythmias occur, which indicates the reliability of the defibrillation process (Fig. 4h).

More sophisticated control of cardiac activity, such as heart pacing, can be achieved through wired contact with a LiDB-powered pacemaker circuit (Supplementary Figs. 12 and 23), which can produce region-specific pacing of heart rhythms (Supplementary Fig. 24, Supplementary Note 2 and Supplementary Video 2). These proof-of-concept demonstrations show the potential clinical utility of LiDBs, for example, to perform low-energy defibrillation for the treatment of heart arrhythmias during cardiac surgery.

### Magnetically maneuverable LiDBs

**Fig. 5:**
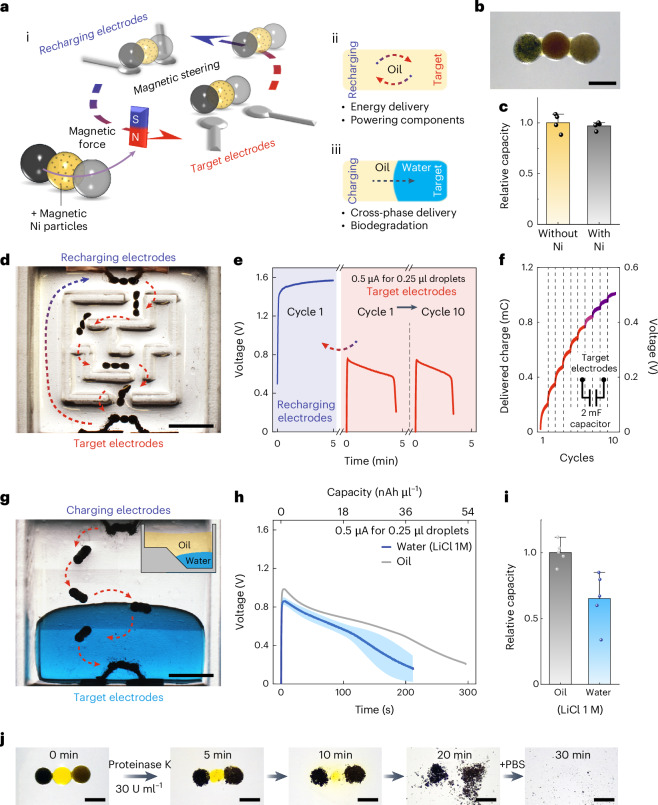
Magnetic propulsion and steering of LiDBs. **a**, The incorporation of magnetic Ni particles in the central separator droplet enabled magnetic manipulation of a LiDB (i). The LiDB can be maneuvered to different target electrodes to perform energy delivery in oil (ii) and from oil into water (iii). **b**, A bright-field image of a LiDB containing Ni particles in the central droplet. **c**, Relative capacities of LiDBs with and without Ni particles. **d**, The trajectory of a LiDB navigating through a maze in oil to perform repeated energy delivery and recharging. **e**, Charge–discharge curves of a LiDB at the first cycle and after the tenth cycle of magnetic navigation. **f**, A 2 mF capacitor was connected to the target electrodes to collect the delivered energy of each cycle. **g**, The trajectory of a LiDB crossing from oil into the aqueous phase (LiCl 1 M, blue dye for visualization). **h**,**i**, Discharge curves (**h**) and relative capacities (**i**) of LiDBs in oil and in water. **j**, Images showing the time sequence of biodegradation after the addition of proteinase K to the aqueous phase.Droplet volumes, 0.25 µl. Scale bars, 800 μm (**b** and **j**) and 3 mm (**d** and **g**). Data in **c**, **h** and **i** are presented as mean values ± s.d. of *n* = 5 replicates. Source data

The hydrogel compartmentalization endowed LiDBs with the ability to incorporate functions in parallel to energy storage. For example, the inclusion of magnetic nickel (Ni) particles in the central separator droplet imparted magnetic maneuverability to LiDBs (Fig. 5a). Ni particles did not affect hydrogel formation (Fig. 5b) or significantly change the battery output (Fig. 5c and Supplementary Fig. 25), yet they allowed manipulation of the entire structure under a magnetic field (Supplementary Videos 3 and 4). By this means, the LiDB can translocate through narrow channels in oil or from oil to an aqueous phase, thereby acting as a mobile energy courier. Figure 5d depicts a LiDB traversing a maze (Supplementary Fig. 26) in oil repeatedly from a pair of recharging electrodes to a pair of target electrodes, where it released the contained energy. The LiDB maintained an over 77% output capacity after ten cycles at 0.5 μA (Fig. 5e). As a proof of concept, a 2 mF capacitor was connected to the target electrodes to collect the delivered charge. The capacitor voltage reached 0.51 V after ten cycles, indicating that 1.02 mC charge (0.26 mJ energy) was delivered by the LiDB (Fig. 5f and Supplementary Fig. 27).

The robust hydrogel composition also allowed the LiDB to release its energy in aqueous media, which is important for biological use but has proved previously challenging for aqueous vesicles/droplets to yield a stable output performance^41^. We magnetically guided a LiDB from oil into aqueous medium containing a pair of target electrodes (Fig. 5g). Upon entering the aqueous phase, the surrounding oil was repelled from the LiDB and replaced by water that contacted the hydrophilic silk hydrogel. The aqueous phase contained 1 M LiCl to prevent Li leakage from the LiDB. For long-term operation, organogel encapsulation^13^ can also be used to prevent electrolyte leakage in a physiological environment (Supplementary Fig. 28). The LiDB functioned in LiCl solution because the output voltage was lower than that required for the electrolysis of water (~1.23 V) (Fig. 5h). The capacity of the battery in LiCl solution was reduced to 67% of that in oil, which was probably due to the hydrogel swelling and, thus, the decrease in surface-to-volume ratio over time (Fig. 5i). We noted that a LiDB cannot be easily propelled back into the oil for recharging because of the strong surface tension of water over the hydrogel. Other mechanisms might be used to assist the transport of LiDBs in aqueous media^42,43^. Once in the aqueous phase, the silk of the LiDB can be enzymatically biodegraded (Fig. 5j), leaving only nanograms of residual materials.

## Discussion

In this work, a miniature soft Li-ion battery was self-assembled from nanoliter hydrogel droplets. The resulting LiDB afforded on-demand activation by UV crosslinking, had a unit volume more than 10^3^-fold lower than previous devices and stored energy at higher density^9–12^. The output energy density of the LiDB made from 10 nl droplets was ~46 μWh cm^−2^, and the instant output power density was ~10 μW cm^−2^, which is sufficient for devices on nanowatt power budgets^3^. These outcomes were facilitated by using a silk hydrogel, with desirable properties including elasticity, Li-ion conductivity, biocompatibility and biodegradability. The LiDB could be interfaced with synthetic tissues to drive charged molecule translocation and with heart tissues to modulate heart activity. The hydrogel droplet construction also permitted the addition of modules, such as one that allows magnetic maneuverability, which may, for example, allow LiDBs to power microrobots for in vivo applications.

There are, however, two caveats concerning our work. First, the electrochemical stability window of water (~1.23 V) limits the output voltage of hydrogel-based batteries in general^44^ to a lower value than that of solid-state batteries^45–47^. Nonetheless, the serial connection of multiple LiDBs can generate higher output voltage to suit application needs. Second, the mass loading of Li particles is limited to less than 20% w/v to prevent nozzle clogging during droplet deposition, thus reducing the maximum battery capacity. The use of smaller Li particles or chemically treated nozzles may prevent clogging and increase the mass loading. Alternative droplet assembly approaches such as acoustic printing^48–50^, microfluidic deposition^51–53^ and droplet self-assembly assisted by functional interlinkers^54–57^ may facilitate scaled-up production of LiDBs and increase the energy density by further decreasing the droplet size. Moreover, our design strategy might be implemented with other hydrogels and aqueous dopants, for example, zinc particles for zinc-ion batteries and iron oxide particles for magnetic control, to build additional members of a class of tiny energy devices that comprise hydrogel, functional particles and conducting elements in soft, miniaturized architectures. The microscale soft LiDB might be used in many fields including microrobots, synthetic tissues, abiotic–biotic interfaces and implantable medical devices.

## Methods

### Materials for LiDBs

All materials except CNT were purchased from Sigma-Aldrich (Merck KGaA). Single-wall CNT dispersion in water was purchased from TUBALL (0.8% wt., BATT H_2_O). Silk fibroin (50 mg ml^−1^) was used to build the hydrogel scaffold. Portions of silk solution were stored at −80 °C before use. For droplet fabrication, silk solution was first left at room temperature for 30 min. Other materials were then added into the thawed silk solution under stirring to form various precursor solutions with the following compositions. Separator solution contained 1 M lithium chloride, 10% v/v poly(ethylene glycol) 400, and 0.5% w/v Pluronic F-68. In addition to the components of the separator solution, the cathode solution contained 30% v/v CNT–water dispersion and 10% w/v lithium manganese(III,IV) oxide (LMO, catalog number 482277); additional components of the anode solution were 30% v/v CNT–water dispersion and 10% w/v carbon-coated lithium titanate (LTO, catalog number 916196). To form various pregels, tris(bipyridine)ruthenium(II) chloride (1 mM final) and sodium persulfate (5 mM final) were added to the precursor solutions and then fully mixed with stirring, while preventing exposure to light. Cathode, separator and anode pregels were kept in black microcentrifuge tubes before droplet deposition and used within 2 h after preparation.

### Lipid/oil solutions

Silk pregel droplets were deposited in a lipid-containing oil and thereby acquired lipid coatings, which subsequently formed lipid bilayers (DIBs) at the interface when droplets were brought into contact. Following previous work^13^, lipids were purchased from Avanti Polar Lipids in powder form and stored at −80 °C. Before use, hexadecane (Sigma-Aldrich) was passed through a 0.22 μm filter (Corning) under vacuum. Lipid films were prepared by bringing ampoules to room temperature and dissolving the lipids in anhydrous chloroform (Sigma-Aldrich) at 25 mg ml^−1^. Using glass syringes (Hamilton), lipid stock solutions, 1,2-diphytanoyl-*sn*-glycero-3-phosphocholine (DPhPC, 90 μl) and 1-palmitoyl-2-oleoyl-*sn*-glycero-3-phosphocholine (POPC, 40 μl), were transferred into a Teflon-capped glass vial (Supelco, 7 ml) that had been cleaned with isopropanol. The chloroform was evaporated under a slow stream of nitrogen while the vial was rotated by hand to produce an even lipid film. The film was dried under vacuum for 24 h and stored under nitrogen at −80 °C before use. When required for droplet fabrication, films were left at room temperature for 30 min, then 1 ml of hexadecane was added to the film, followed by sonication (Branson 2800) for 1 h. The total concentration of lipids was 4 mM with a molar ratio of DPhPC:POPC of 2:1. Lipid films were kept for a maximum of 2 months.

### LiDB fabrication and activation

Droplet deposition was carried out in a dark room under a noncrosslinking yellow/red light. Droplets were formed in custom-made hydrophobic glass wells that contained 200 μl of lipid-containing oil. Droplets of pregel solution were placed in each well with a programmable microinjector (FemtoJet, Eppendorf), which ejected droplets from a loaded glass nozzle (Femtotips, Eppendorf) with volumes that ranged from femtoliters to microliters. Single LiDBs were obtained by depositing droplets so that they contacted one another, allowing lipid bilayers to form at the interfaces, which happened within seconds. After formation, LiDBs could be drawn by capillary action into a truncated pipette tip along with surrounding oil, and then placed in other wells for further characterization and applications. After fabrication, LiDBs could be stored for more than 2 days in the dark within a lipid/oil solution in a humid incubator without energy dissipation due to the insulating DIBs. To activate LiDBs, the droplets were exposed to UV light (365 nm, power density >0.5 mW cm^−2^, Thorlab) for 60 s to photochemically crosslink the silk fibroins, which disrupted the DIBs and led to the formation of a continuous silk hydrogel structure.

### Characterization

A custom-made measurement system containing two 3D manual micromanipulators (NMN-21, Narishige) and an integrated microscope (Stereo Zoom 5, Leica) was used to examine the microscale LiDBs. We used carbon fiber electrodes (Goodfellow) to contact the cathode and anode compartments of an LiDB. Gold electrodes (50 μm, Sigma-Aldrich) could also be used for smaller droplets. The electrochemical performances of LiDBs were tested with an electrochemical workstation (μStat-i 400, Metrohm DropSens), which included cyclic voltammetry, galvanostatic charge–discharge measurement, amperometric detection, electrochemical impedance analysis and alternating current impedance spectra. To make a three-electrode system, Ag/AgCl electrodes and excessive activated carbon were used as reference and counter electrodes (DRP-11L-U75, Metrohm). Fourier-transform infrared spectra were measured in attenuated total reflection mode (Bruker Tensor 27 spectrometer). Blank background measurements were subtracted from each spectrum. The zeta potential was measured by a Malvern Zetasizer (Nano-ZS). The compression response was measured by an electromechanical test system (Criterion, MTS Systems).

### Powering electronics with LiDBs

Several LiDBs were moved into a custom-made transparent resin well, which was fabricated by a 3D printer (Formlabs, Solid Print3D) and had embedded screen-printed carbon electrodes (CNT paste, Nanoshel) on the well bottom. Six LiDBs were connected in series over the carbon electrodes to increase the output voltage. Red light-emitting diodes (HLMP-K150, Broadcom), a liquid-crystal display timer (Sigma-Aldrich) and a pulse generator circuit based on a 555-timer chip (TLC555IP, RS PRO) could all be powered by the six LiDBs. Capacitor (RS PRO) charging curves were measured by a Keithley 617 programmable multimeter set to voltage measurement mode with high input impedance (~2 TΩ). An oscilloscope (PicoScope 2000) was used to measure the generated pulses.

### Powering the translocation of molecules

Hydrogel droplets were prepared by dissolving low-gelling-temperature agarose powder (2% w/v, Sigma-Aldrich) in 0.1 M potassium chloride (Sigma-Aldrich) and heating the solution to 90 °C. The solution was then cooled down to 37 °C before adding other materials. Cationic pyronin Y (10 μM, Sigma-Aldrich) and anionic MANT-dATP (100 μM, Thermo Fisher Scientific) were used as fluorescent markers. Aqueous synthetic cells contained 0.1 M potassium chloride, 0.5% w/v Pluronic F-68 (Sigma-Aldrich), and 50 μg ml^−1^ of the pore-forming protein αHL (with the mutation T129C, provided by X. Wang (Bayley Group). The protein was expressed in *Escherichia coli* cells, purified as monomers (confirmed by SDS–PAGE electrophoresis) and stored at −80 °C. Aqueous synthetic cells were deposited in lipid-containing hexadecane (4 mM lipids at a molar ratio of DPhPC:POPC of 2:1); DIBs formed between each synthetic cell. The converting hydrogel droplets (Fig. 3a) contained 2% w/v low-gelling-temperature agarose, 0.1 M potassium chloride and 1.1% w/v PEDOT:PSS (neutral pH, Sigma-Aldrich). Converting droplets were attached one to the cathode and one to the anode of a LiDB in lipid-free oil, creating a continuous hydrogel LiDB device. The hydrogel droplets were submerged in lipid-free oil, and the aqueous synthetic cells were submerged in lipid-containing oil. To enable charged molecule translocation, the LiDB device was transferred into a well containing the synthetic cells, where the LiDB device was attached to the synthetic cells through its termini to form a closed circle.

### Optical recordings

Bright-field images were recorded with a stereomicroscope (EZ4 W, Leica) or a wide-field stereomicroscope (SMZ660, Nikon). A Leica DMi8 inverted epi-fluorescence microscope was used for fluorescence imaging. Images were processed using the Leica Application Suite X and Fiji (ImageJ). Fluorescence intensities were obtained by using the Fiji freehand tool, intensity measurements and the profile plots tool. The hydrogel morphologies were characterized by scanning electron microscopy (HITACI S4800). All hydrogel droplets were first frozen with liquid nitrogen and then freeze-dried by a vacuum freeze dryer to completely drain the water.

### Biocompatibility tests with LiDBs

All cells were seeded into 96-well plates and cultured for 3 days to form uniform layers before testing. Single fully charged LiDBs formed from 1 μl droplets were placed on to the cells in each well and formed close contact with them through the adhesive silk hydrogel. 3T3 fibroblasts (NIH-3T3, CRL-1658) expressing green fluorescence protein were used for live/dead imaging. The LiDB was left in the well with the cells during 48 h of culture before imaging. Six wells were randomly picked and examined in detail. The cells were incubated with 5.0 μM propidium iodide (Sigma-Aldrich) for 60 min at 37 °C before imaging with a fluorescence confocal microscope (SP5, Leica). *Z*-stack images were acquired between the bottom of the cells to 100 μm above them with a step of 2 μm per image. Maximum *Z* projection was then conducted to generate final images, which were processed using the Leica Application Suite X. PrestoBlue assays (Thermo Fisher Scientific) were used to determine live cell numbers and viability. The cells were incubated with 10% v/v PrestoBlue reagents for 60 min at 37 °C before fluorescence measurement. A microplate reader (CLARIOstar Plus) was used to quantify the fluorescence and, hence, the number of living cells. Further, human dermal fibroblasts (Lonza) and atrial and ventricular cardiomyocytes differentiated from Kolf 2.1J human iPS cells^58^ (The Jackson Laboratory) were used to quantify the effects of LiDBs on cell metabolic activity, cytotoxicity and apoptosis. Cell metabolic activity was measured by using an 3-(4,5-dimethylthiazol-2-yl)-2,5-diphenyltetrazolium bromide (MTT) cell proliferation assay kit (Vybrant, Thermo Fisher Scientific). Cytotoxicity was measured by using a cell cytotoxicity assay kit (Colorimetric, ab112118, Abcam). Apoptosis was measured by using a caspase assay kit (Caspase-Glo 3/7 Assay, Promega). Standard assay protocols were followed after cells had been co-cultured with LiDBs for 7 days.

### Ex vivo heart preparation and ECG monitoring

Animal experiments were performed on mice in accordance with the UK Animals Scientific Procedures Act (1986) and were approved by the University of Oxford Pharmacology Ethics Committee under project licence (PP8557407). All procedures were performed in conformity with national and institutional guidelines and regulations. All mice including *Pnmt*^*Cre/ChR2*^ mutant mice^40^ and wild-type (WT) littermates used in this study were maintained in pathogen-free facilities at the University of Oxford. Mice were given ad libitum access to food and water. WT mice and genetically modified *Pnmt*^*Cre/ChR2*^ mice were killed by cervical dislocation (schedule 1 killing). The heart was immediately removed and cannulated onto the Langendorff system at the aorta to allow retrograde perfusion of Kreb’s solution, which contained 128 mM sodium chloride, 4.8 mM potassium chloride, 22 mM sodium bicarbonate, 11 mM glucose, 1 mM magnesium chloride and 1.8 mM calcium chloride. The solution was bubbled with 95% oxygen and 5% carbon dioxide for at least 15 min before use. The perfusion medium was held at 37 °C with a flow rate of 3.5 to 4 ml min^−1^. To monitor cardiac rhythms, we carried out ex vivo ECG analysis on the Langendorff-perfused hearts. In addition to the metal cannula, two electrodes were placed adjacent to the right atrium and the bottom left ventricle. The ECG was filtered with low and high cutoff frequencies of 5 and 500 Hz, digitized at a sampling frequency of 1 kHz (1401, Cambridge Electronic Design), and recorded by the Spike 2 software (Cambridge Electronic Design). ECG parameters including RR interval, PR interval, QRS and QT durations were monitored.

### Optogenetic stimulation on ex vivo heart

Langendorff-perfused ex vivo hearts from *Pnmt*^*Cre/ChR2*^ mutant mice were subjected to programmed light stimulation through the activation of ChR2 light-sensitive channels. This was achieved by the delivery of 470 nm, 5 ms blue-light pulses generated by OptoFlash (Cairn Research). The pulses were triggered by a 1401 digitizer and Spike 2 software (Cambridge Electronic Design). The blue light intensity was tuned with an 818-ST2 Wand Detector connected to an 843R Power meter (both Newport Corporation). ECGs indicative of left atrial pacing were recorded from *Pnmt*^*Cre/ChR2*^ hearts when light pulses were delivered to the left atrium.

### Heart stimulation by LiDBs

LiDBs were fully charged for 10 min at a current of 0.5 μA and immediately used for stimulation to ensure battery output consistency. To quantify direct contact stimulation, *Pnmt*^*Cre/ChR2*^ hearts were used under light-regulated pacing to prevent ectopic heartbeats. A single LiDB was drawn into a truncated pipette tip by capillary action and gently placed on the right side of the heart for each stimulation. Because of the adhesive silk hydrogel, LiDBs attached to the heart surface without falling off. To remove the LiDB, we gently pipetted it away or rinsed it off by using perfusion medium. To create heart fibrillation, we perfused 300 μl of 0.3 mg ml^−1^ ouabain solution (Sigma-Aldrich) into the hearts and used high-frequency wired burst pacing to trigger ventricular arrhythmias. Once ECG signals denoted the beginning of heart fibrillation, we stopped the high-frequency pacing. Heart fibrillation could last for minutes and eventually lead to permanent heart death. We used LiDBs to produce electrical defibrillation in the same manner as the direct contact stimulation.

For the wired stimulation, we used a LiDB power pack of typically six LiDBs in series to drive a home-built pulse generator. The pulse width was ~5 ms, and the pacing frequency could be tuned between 1 Hz and 15 Hz. A pair of Ag/AgCl stimulating electrodes was placed on the right atrium (near the sinoatrial node) or on any region of the ventricles of WT mouse hearts to produce pacing pulses.

### Magnetically controlled energy delivery and recharging

To form a magnetically responsive silk hydrogel, the separator solution contained additional 10% v/v nickel particles (MagneHis, Promega). LiDBs were then fabricated as before. A neodymium magnet (N42, first4magnets) placed at ~2 cm above a LiDB was used to guide movement. The magnet was mounted on a 3D manual micromanipulator (NMN-21, Narishige) for precise navigation. The maze and the double-deck well were fabricated by a 3D printer (Formlabs, Solid Print3D) and featured embedded screen-printed carbon electrodes (CNT paste, Nanoshel) on the well bottom. Copper tape (RS PRO) was used to facilitate connection between the screen-printed carbon electrodes and measurement wires/clips.

### Biodegradation of LiDBs

LiDBs were transferred into wells containing 200 μl PBS (pH 7.4, Gibco) with 30 U ml^−1^ (1 mg ml^−1^) proteinase K (Thermo Fisher Scientific) at 37 °C. LiDBs were enzymatically degraded for 20 min (six repeats), precipitating CNT and LMO/LTO particles, which sank to the bottoms of the wells.

### Statistics and reproducibility

Statistical analyses were performed using Origin, and *P* values were determined by unpaired two-sided analysis of variance. Six, five and five independent mouse hearts were used for the shock, defibrillation and pacing experiments, respectively. Each experiment used a minimum of three independent LiDBs. No data were excluded from the analyses.

### Reporting summary

Further information on research design is available in the Nature Portfolio Reporting Summary linked to this article.

## Supplementary information

Supplementary Information Supplementary Notes 1 and 2, Figs. 1–28, Table 1 and References.

Reporting Summary

Supplementary Video 1 Ex vivo murine heart stimulation by the LiDB. Direct contact by a LiDB to produce electrical shock/defibrillation.

Supplementary Video 2 Ex vivo murine heart pacing by LiDBs. Wired contact for pacing. We used a LiDB power pack of six LiDBs in series to drive a home-built pulse generator. The pulse width was ~5 ms, and the pacing frequency was ~10 Hz.

Supplementary Video 3 Magnetically maneuverable LiDBs (speed 0.5×). The LiDB was propelled and steered under a magnetic field. The play speed of the video is half of the recording speed (slow motion mode).

Supplementary Video 4 Magnetically maneuverable LiDBs (speed normal). The LiDB was propelled and steered under a magnetic field. The video is at a normal play speed.

## Source data

Source Data Fig. 2 Statistical source data.

Source Data Fig. 3 Statistical source data.

Source Data Fig. 4 Statistical source data.

Source Data Fig. 5 Statistical source data.

## Data Availability

All data generated during this study are included in the Article and its Supplementary Information. Data are also available from the corresponding authors on request.
