# Supplementary material for: A microscale soft lithium-ion battery for tissue stimulation
Source: Nat Chem Eng. 2024 Oct 25;1(11):691–701. doi: 10.1038/s44286-024-00136-z (PMC11606923; doi:10.1038/s44286-024-00136-z)
Supplement: Supplementary file 1 — Supplementary Notes 1 and 2, Figs. 1–28, Table 1 and References. [file 44286_2024_136_MOESM1_ESM.pdf]

nature chemical engineering

Supplementary information https://doi.org/10.1038/s44286-024-00136-z

A microscale soft lithium-ion battery for tissue stimulation

In the format provided by the authors and unedited

Table of contents

Supplementary Notes 1 and 2……………………………………………………………………...…2

Supplementary Figs. 1 to 28…………………………………………………………………...…4

Supplementary Table 1………………………………………………………………………….33

References………………………………………………………………………………………..34

Supplementary Note 1.
Volume and internal resistance of droplets

The contact area is important in determining the droplet's internal resistance and the volumetric capacity. After stabilization, the contact area (A) between two droplets can be expressed in terms of the contact angle θ and the radius of curvature r of the adhered droplets:

$$A = \pi r^2 \sin^2 \theta \quad \text{(Eq. 1)}$$

where θ is determined by the compositions of the lipids, oil and pre-gel solutions[1]. The internal resistance (R) of lithium-ion droplet batteries (LiDBs) depends on the droplet volume, which is directly proportional to the length of the ion conductive pathway (l) and inversely proportional to the average cross-sectional area ($S_{average}$) of the droplet. Relating l and $S_{average}$ to r and A of the droplets, we obtain:

$$R = \frac{\rho l}{S_{average}} \approx \frac{6\rho r}{A} = \frac{6\rho}{\pi r \sin^2 \theta} \quad \text{(Eq. 2)}$$

where ρ is the resistivity of the silk hydrogel. Therefore, as the volume and hence the contact area decrease, the internal resistance of LiDBs will increase and the output current (i.e., the short-circuit current) will decrease. However, decreasing the droplet volume will increase the droplet surface-to-volume ratio, which facilitates the electrode reactions and enhanced the coulombic efficiency and volumetric capacity[2,3].

Supplementary Note 2.

***Ex vivo* murine heart pacing powered by LiDBs**

LiDBs can generate cardiac pacing by powering a pacemaker circuit through wired contact (Supplementary Fig. 23 and 24). Wired contact through an electrical pulse generator promises sophisticated output features that yield regional specific control of cardiac electrical activity. As an example, heart pacing with different pulsing frequencies was mediated by a LiDB power pack and a pacemaker circuit (Supplementary Fig. 24a). The electrical energy generated from 6 LiDBs was stored in the pacemaker circuit, whose output was connected to the heart by a pair of pacing electrodes (Supplementary Fig. 24b and Supplementary Video 2). Multiple LiDBs had sufficient output intensity to overdrive the intrinsic sinus rhythm (Supplementary Fig. 24c) and generate both atrial and ventricular pacing: The pacing region was located at the right atrium (near the sinoatrial node) to achieve atrial pacing (Supplementary Fig. 24d) or at any region of ventricles to conduct ventricular pacing (Supplementary Fig. 24e), showing two different epicardial electrocardiogram (ECG) patterns. The frequency of the stimulation pulse was tuned between 10 and 2.5 Hz with a fixed pulse width of ~5 ms (Supplementary Fig. 24f). When the pacing stimuli were faster than (e.g., 10 Hz) or near (e.g., 3.5 Hz) the intrinsic heart rhythm (~4 to 5 Hz), the electrical pulses induced contraction and regulated the heart rate. Pulses with a lower frequency (e.g., 2.5 Hz) were not able to override the intrinsic heart rhythm, resulting in a mixed ECG signal reflecting both paced and intrinsic heartbeats. One LiDB power pack could power 3 stimulation cycles with each cycle lasting for ~15 s. In each cycle, with the consumption of the energy by pacing, the pulse frequency declined in the last ~4 s, leaving an invalid pacing ECG at the end. We quantified the proportion of valid pacing during 3 independent cycles (Supplementary Fig. 24g). By setting the selection range of ±20% of the pacing frequency, 64% of paced beatings were valid with frequencies larger than 8 Hz for the 10 Hz pacing (425 pulses in total), and 43% beatings were valid with frequencies lower than 3 Hz for the 2.5 Hz pacing (288 pulses in total). This level of working duration was comparable to other reported implantable power sources as to perform first-aid and short-term biomedical functions[4,5], and might be further prolonged by using more LiDBs in the power pack. Further electronic engineering of the pulse generator circuit based on the outputs of LiDBs could also prolong the stimulation time.

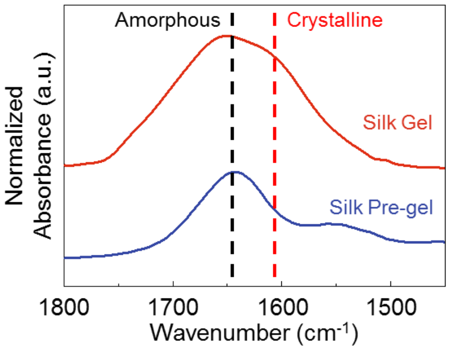

Supplementary Fig. 1 Infrared spectroscopy of silk pre-gel (blue) and UV-crosslinked hydrogel (red). Absorption assigned to the crystalline region increased due to the formation of β-sheet. Three absorption bands have been proposed at 1625, 1645, and 1660 cm^{-1}, associated with β-sheet, random coils, and α-helices, respectively[6,7]. Samples were analyzed by using Fourier-transform infrared spectroscopy in attenuated total reflection mode.

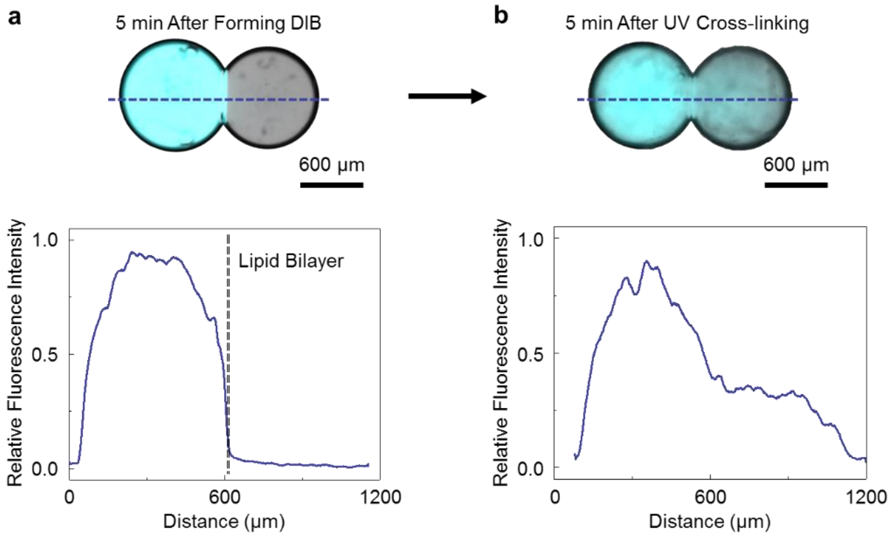

Supplementary Fig. 2 Rupture of the lipid bilayer between two pre-gel droplets. After UV-crosslinking of the silk hydrogel, a continuous hydrogel was formed. Subsequently, the bilayer-impermeable dye ATTO-488 (1 μM, Sigma-Aldrich) diffused into the adjacent droplet, indicating bilayer rupture. Overlaid bright-field and fluorescence microscopy images (top) and relative fluorescence intensity (bottom) of a droplet pair before (**a**) and after (**b**) formation of a continuous hydrogel.

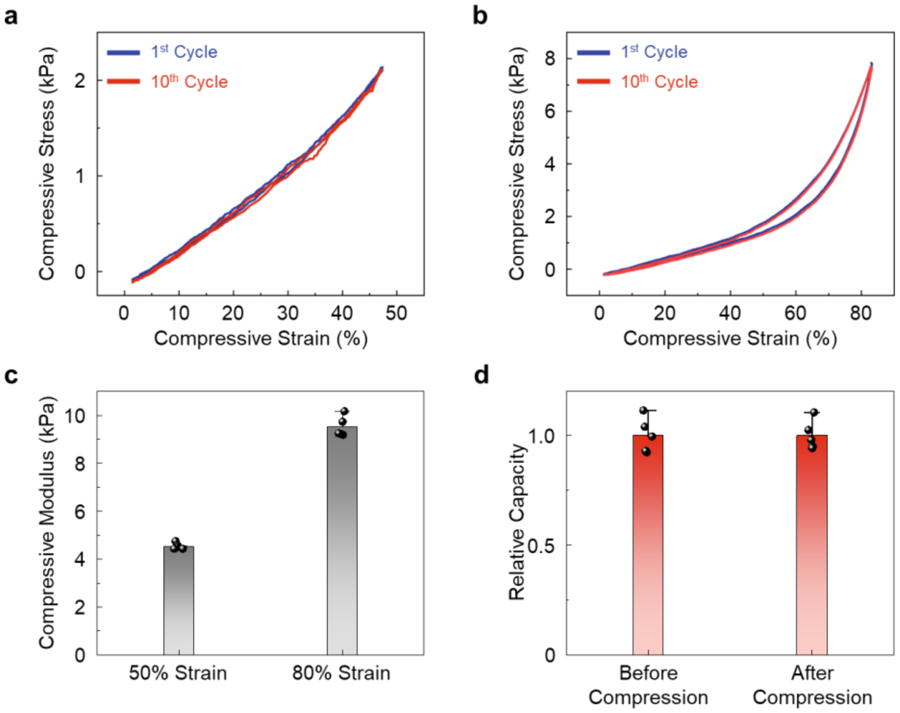

Supplementary Fig. 3 Compression responses of LiDBs. a and **b**, Cyclic compressive stress-strain curves of LiDBs under 0–50% (**a**) and 0–80% strain (**b**). **c**, Compressive modulus as a function of compressive strain. **d**, Relative capacities of LiDBs before and after 80% compressive strain. Data in **c** and **d** are presented as mean values ± standard deviations (s.d.) of n = 5 replicates.

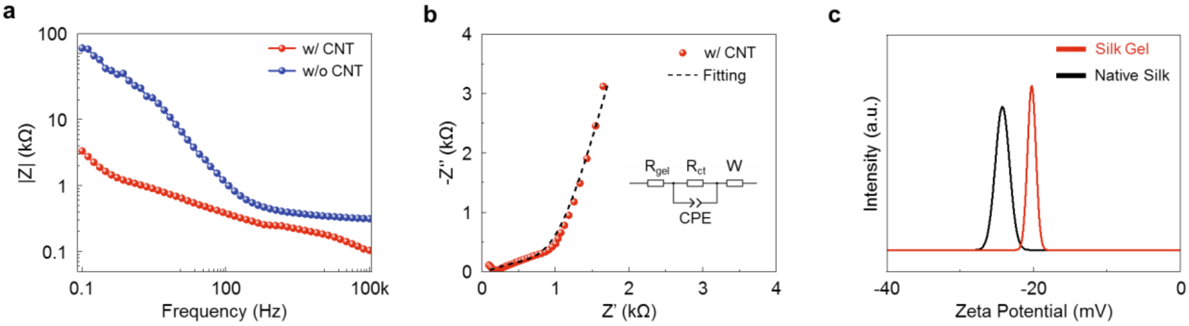

Supplementary Fig. 4 Electrochemical impedance spectroscopy and zeta potentials of silk hydrogels. a, Alternating current impedance spectra over a frequency range from 0.1 to 100 kHz. The electrical conductivity of the silk hydrogel was calculated by Ohm's law. The silk hydrogel with CNT had an electrical conductivity of ~41.3 mS cm^{-1}, which was 30-fold higher than the silk hydrogel without CNT (~1.3 mS cm^{-1}). **b**, Theoretical fitting of the silk hydrogel with CNT. Inset, equivalent circuit. Charge transfer resistance (R_{ct}) of the CNT–Li-particles–electrolyte interface was ~1025 Ω. **c**, Zeta potentials of the native silk solution and silk hydrogel diluted at 2.5 mg mL^{-1}.

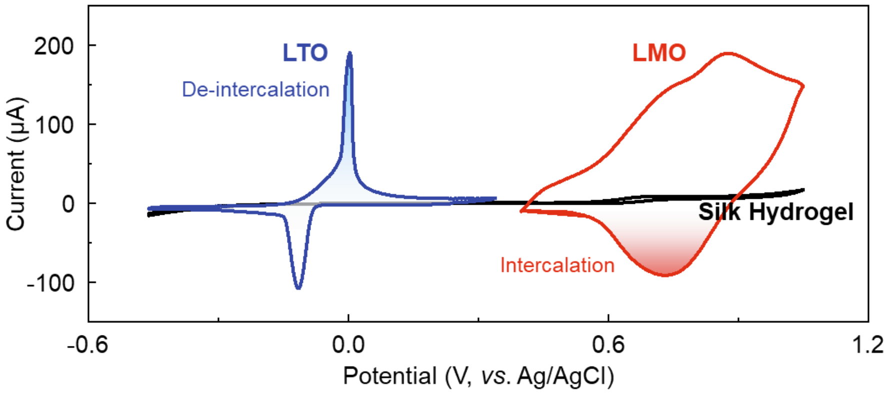

Supplementary Fig. 5 Cyclic voltammograms of the silk hydrogel (black), silk-LTO (blue) and silk-LMO (red) hydrogels at scan rates of 10 mV s^{-1}. A mass loading of 10% w/v LTO or LMO in the silk hydrogel was used.

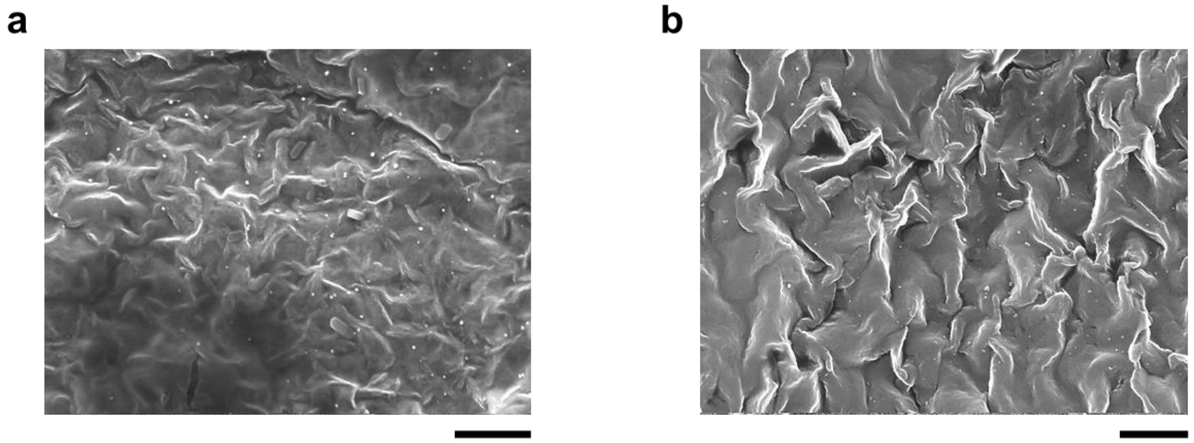

Supplementary Fig. 6 Scanning electron microscope images of the dehydrated silk-LMO (a) and silk-LTO (b) hydrogels, showing uniform distributions of Li particles and CNT. A volume loading of 30% v/v CNT and a mass loading of 10% w/v LMO or LTO in the silk hydrogel were used. Scale bars, 1 µm.

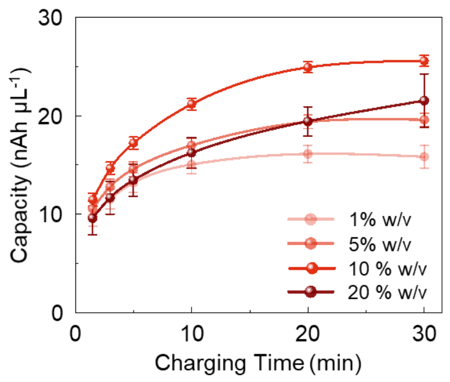

Supplementary Fig. 7 Volumetric capacities of LiDBs with different mass loadings of LMO in the cathode droplet and LTO in the anode droplet. Data are presented as mean values ± s.d. of n = 3 replicates.

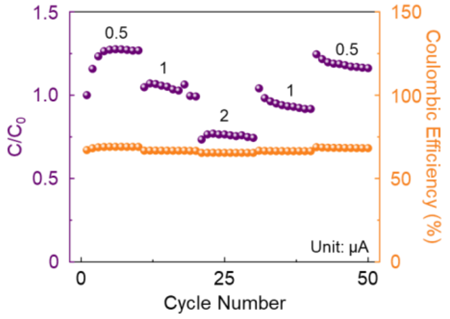

Supplementary Fig. 8 Cyclic performance and rate capability of LiDBs at charge-discharge current of 0.5, 1, and 2 μA. C_0 and C correspond to volumetric capacities before and after the cycle.

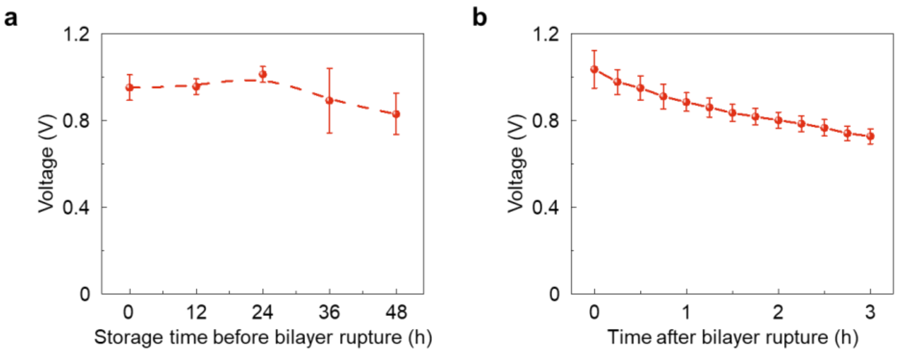

Supplementary Fig. 9 Voltage-time curves measured for the LiDB. a, Output voltages of LiDBs after different storage times in oil before activation. **b**, Self-discharge of the LiDB after activation by formation of a hydrogel structure. Data are presented as mean values ± s.d. of n = 5 replicates.

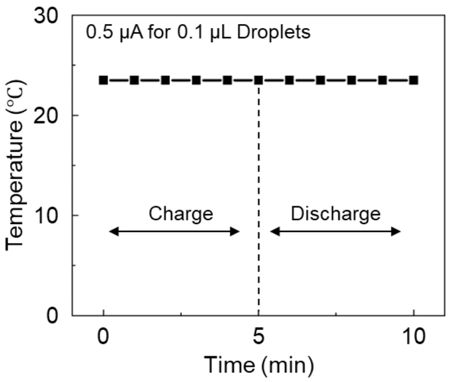

Supplementary Fig. 10 Temperature of a LiDB at a charge-discharge current of 0.5 μA. A temperature sensor with a probe size of less than 1 mm^2 was placed underneath the LiDB. 0.1 μL droplets were used.

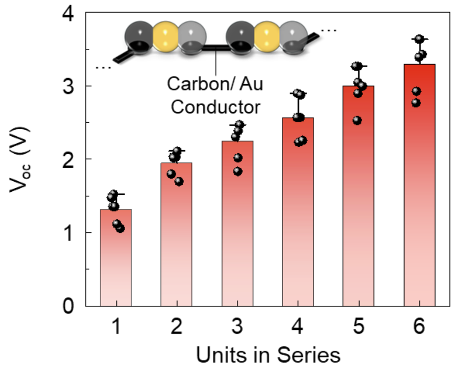

Supplementary Fig. 11 Normalized open circuit voltage of LiDBs connected in series by underneath screen-printed carbon electrodes. 0.5 μL droplets were used. Data are presented as mean values ± s.d. of n = 5 replicates.

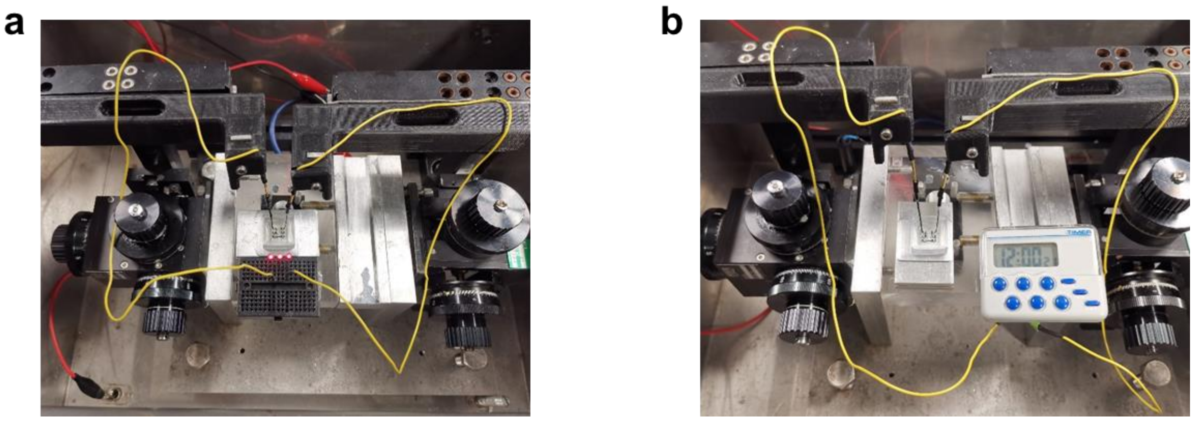

Supplementary Fig. 12 6 LiDBs were connected to light up three red light-emitting diodes (a) **and a liquid-crystal display timer** (b)**.**

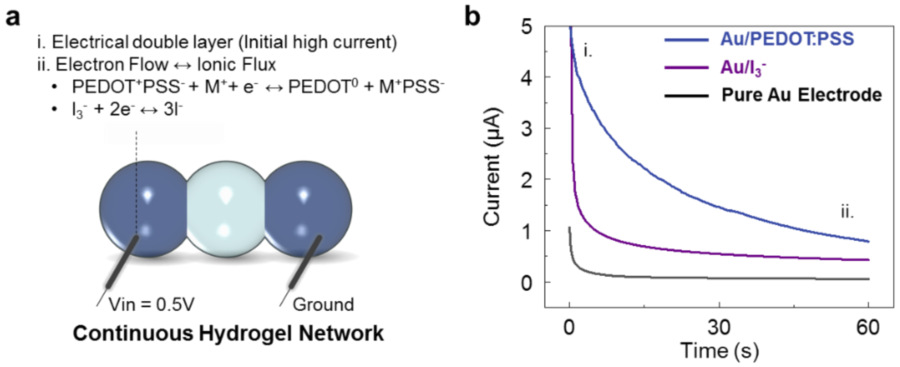

Supplementary Fig. 13 Measurement of the converting hydrogel droplets. a, Agarose droplets (2% w/v, 0.5 μL) containing 100 mM potassium chloride were used. The two terminal droplets (dark blue) contained the redox materials, such as poly(2,3-dihydrothieno-1,4-dioxin)-poly(styrenesulfonate) (PEDOT:PSS, 1.1% w/v) or I_3^- (0.6 M KI, 0.4 M I_2). The converting droplets were used to convert the electron flow from connected electrodes into the ionic flux in the central hydrogel droplet (light blue). When applying an input voltage on the electrodes, the ions in the converting droplets first formed electric double layers surrounding the electrodes (**i**), creating an initial current peak. Then, redox reactions occurred due to the presence of the redox materials in the converting droplets (**ii**). **b**, Measuring the current with gold electrodes by applying 500 mV input voltage. The redox materials can produce more ion flux and thus provide a higher current by comparison with pure electrodes. Although KI/I_2 (Lugol solution, Sigma-Aldrich) could conduct a redox reaction, the large solubility of I_2 in oil limits its applicable time. Therefore, we used PEDOT:PSS for converting.

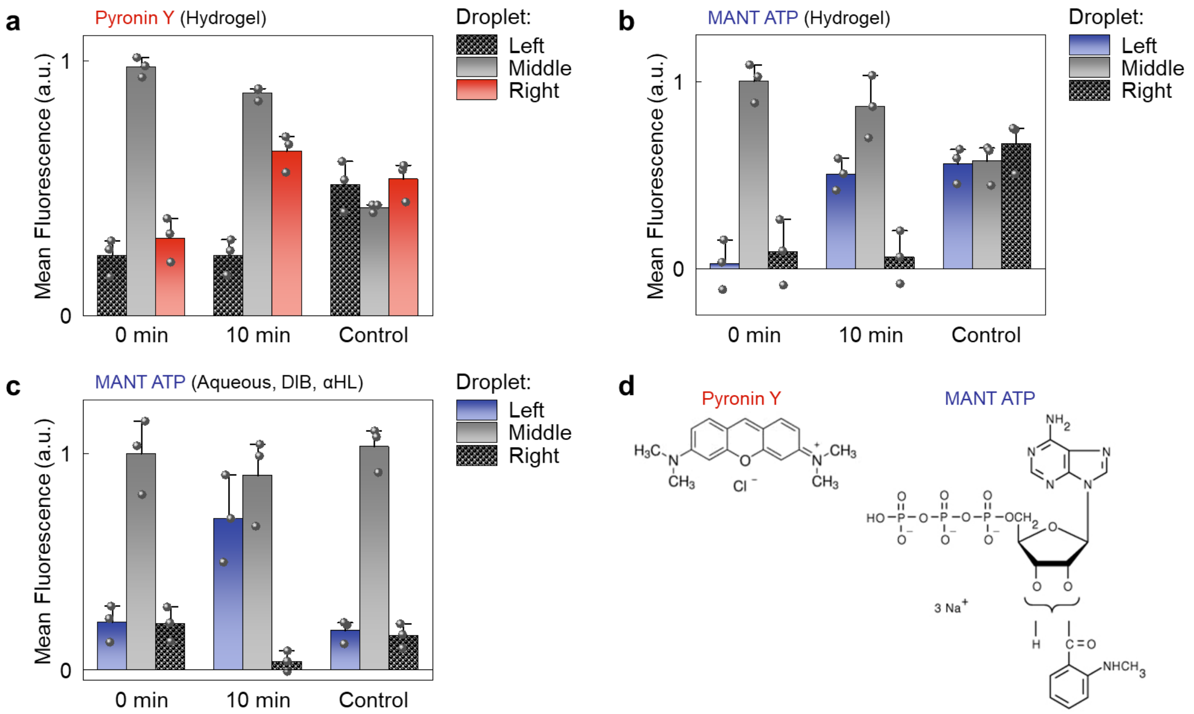

Supplementary Fig. 14 Relative fluorescence of the three connected droplets before and after charged molecule translocation powered by the LiDB. a, Cationic pyronin Y (10 μM) moved toward the negative anode of the LiDB (the right droplet). **b** and **c**, Anionic MANT-dATP (100 μM) moved toward the positive cathode of LiDB (the left droplet) in hydrogel droplets (**b**) and aqueous (**c**) synthetic cells. **d**, Molecular structures of pyronin Y and MANT-dATP. Control group stands for experiments using fully discharged LiDBs. Data are presented as mean values ± s.d. of n = 3 replicates.

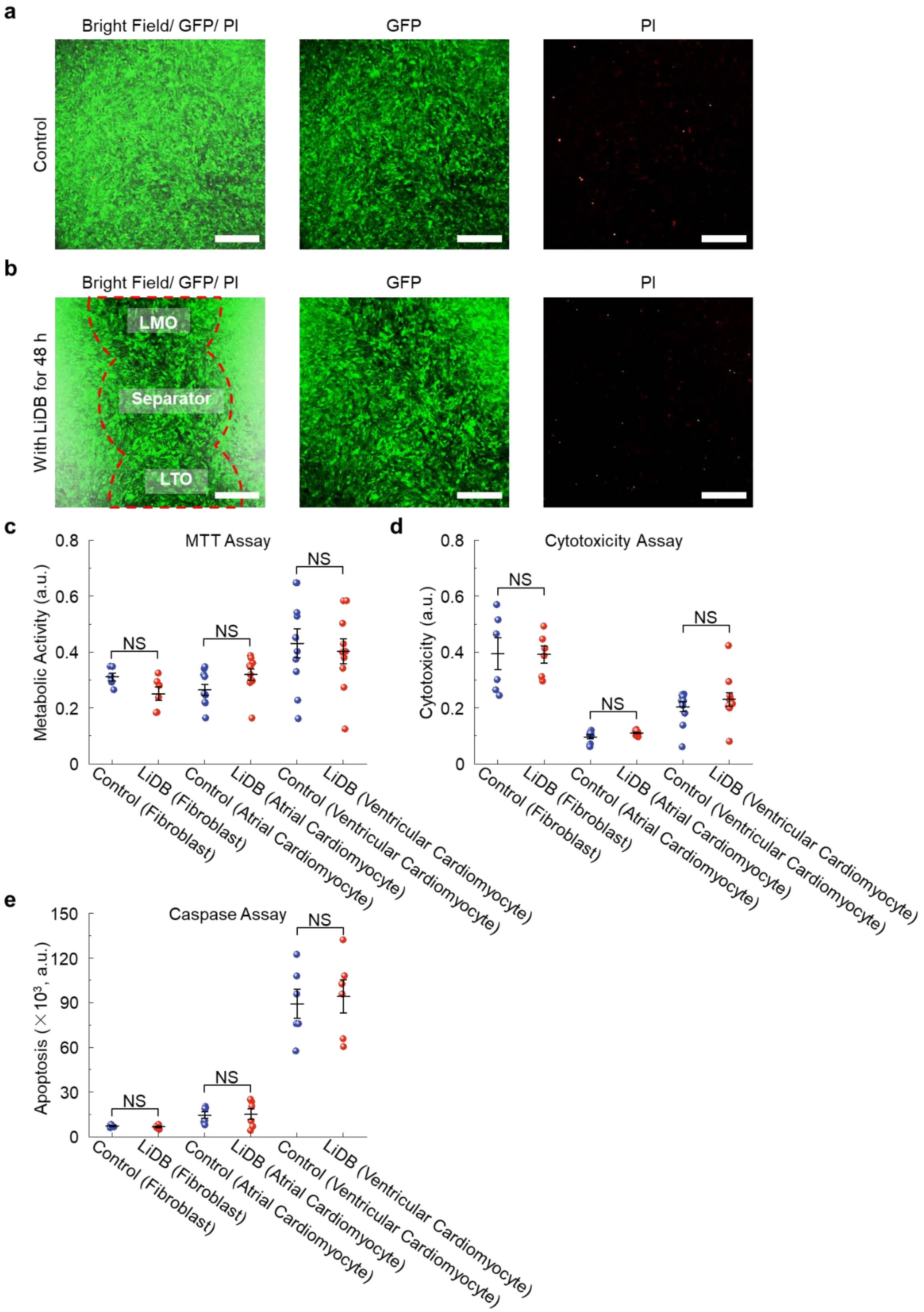

Supplementary Fig. 15 Biocompatibility of LiDBs. a and **b**, Live/dead imaging of 3T3 fibroblasts shows the live (green) and the dead cells (red). The control group was not contacted with a LiDB (**a**). Cells in experimental group were subjected to LiDBs with 1 μL

droplets (**b**). Red dashed line denotes the outline of the attached LiDB. Cells were attached with LiDBs for 48 hours before imaging. Scale bars, 300 μm. **c**–**e**, Cell assays with human fibroblasts, or atrial or ventricular cardiomyocytes (derived from human iPSCs), after 7 days of co-culture with LiDBs. MTT assays reveal cell metabolic activity and viability (**c**). Cytotoxicity assays monitor cytotoxic effect of LiDBs (**d**). Caspase assays detect caspase activity and cell apoptosis (**e**). Data are presented as mean values ± s.d. of n = 6, 10 and 10 replicates for fibroblasts, atrial and ventricular cardiomyocytes, respectively. NS, not significant; two-sided t-test.

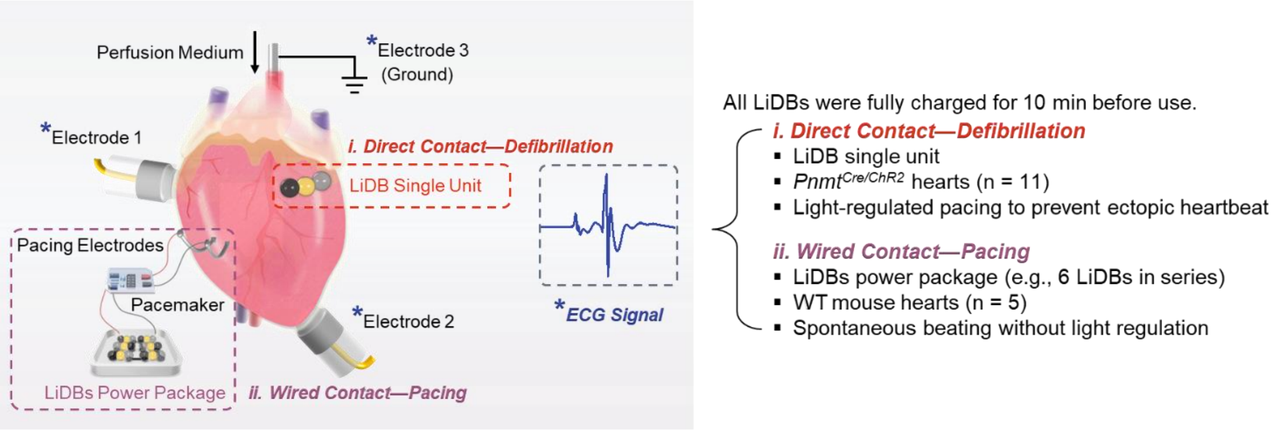

Supplementary Fig. 16 Schematic to illustrate the two different stimulation approaches powered by LiDBs.

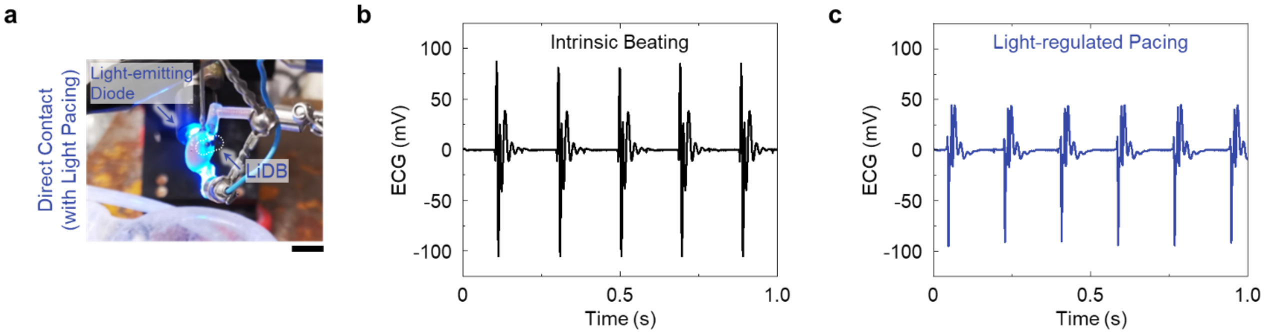

Supplementary Fig. 17 Optogenetic control of heart rhythm by blue light pacing. a, Image of the pacing set up. Scale bar, 1.2 cm. **b**, ECG of the intrinsic heartbeats. **c**, ECG under light-regulated pacing to avoid ectopic heartbeats.

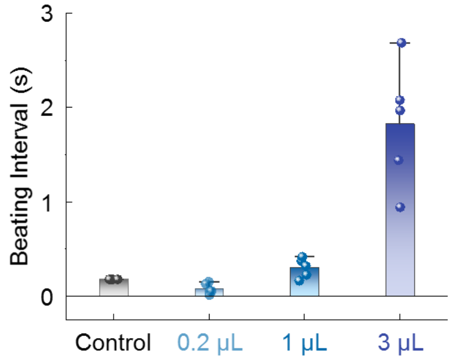

Supplementary Fig. 18 Comparison of induced beating intervals of LiDBs with different droplet volumes. Control group stands for fully discharged LiDBs (intrinsic heartbeats). Data are presented as mean values ± s.d. of n = 5 independent mouse hearts.

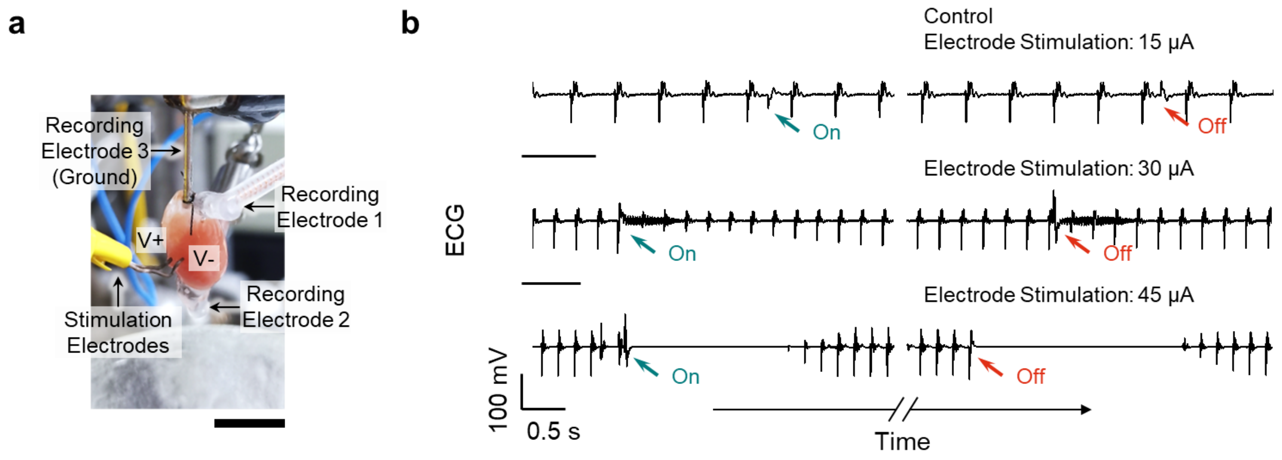

Supplementary Fig. 19 ECG traces of hearts under direct current stimulation through wired electrodes. a, Image of the electrode stimulation. Scale bar, 1 cm. **b**, Green arrows mark the times of stimulation on and red arrows mark the times of stimulation off. Stimulation by a 30 μA direct current was equivalent to the application of a LiDB with 1 μL droplet volume, and showed a similar shock signal, heartbeat suppression, and subsequent restoration in the ECG trace.

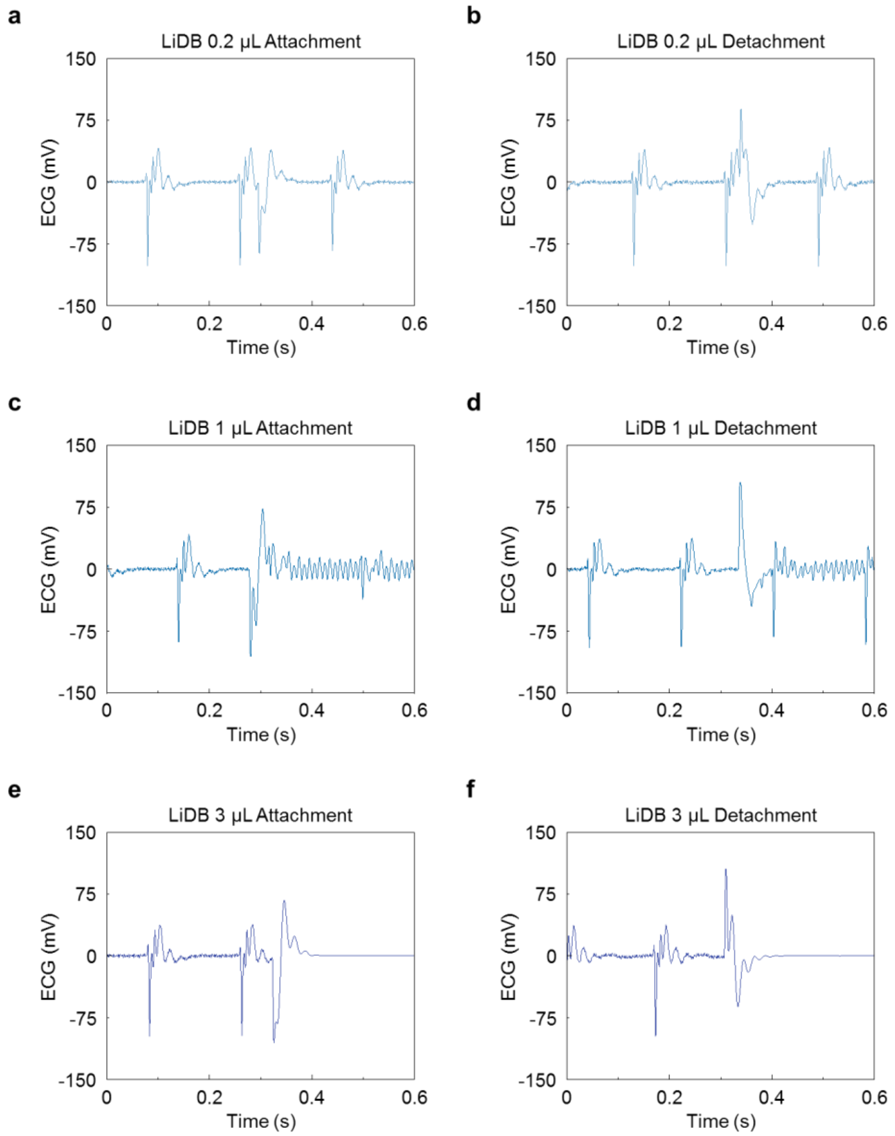

Supplementary Fig. 20 ECG traces of hearts at the times of LiDB attachment and detachment. Different extents of electrical stimulation were produced by using LiDBs with droplet volumes of 0.2 (**a** and **b**), 1 (**c** and **d**), and 3 μL (**e** and **f**).

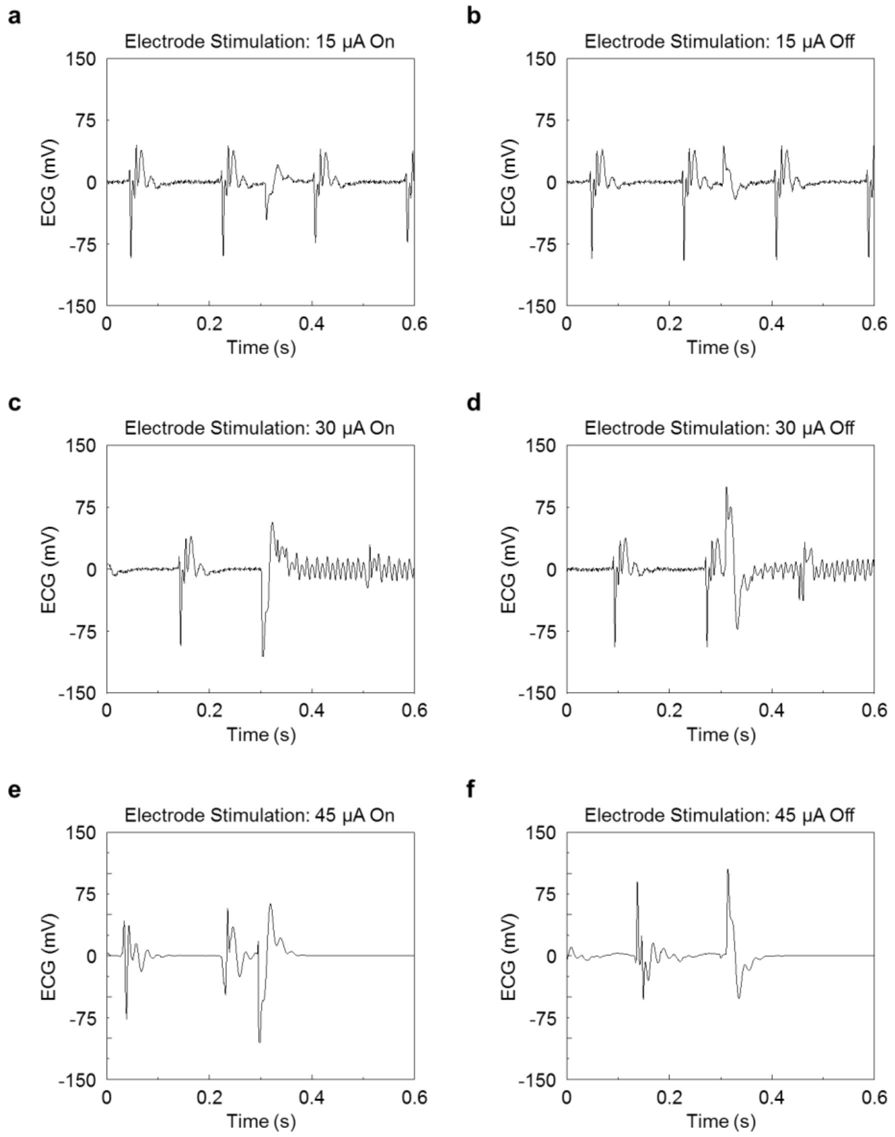

Supplementary Fig. 21 ECG traces of hearts at the beginning and ending times of constant direct current stimulation through wired electrodes. Different extents of electrical stimulation were produced by applying currents of 15 (**a** and **b**), 30 (**c** and **d**), and 45 µA (**e** and **f**).

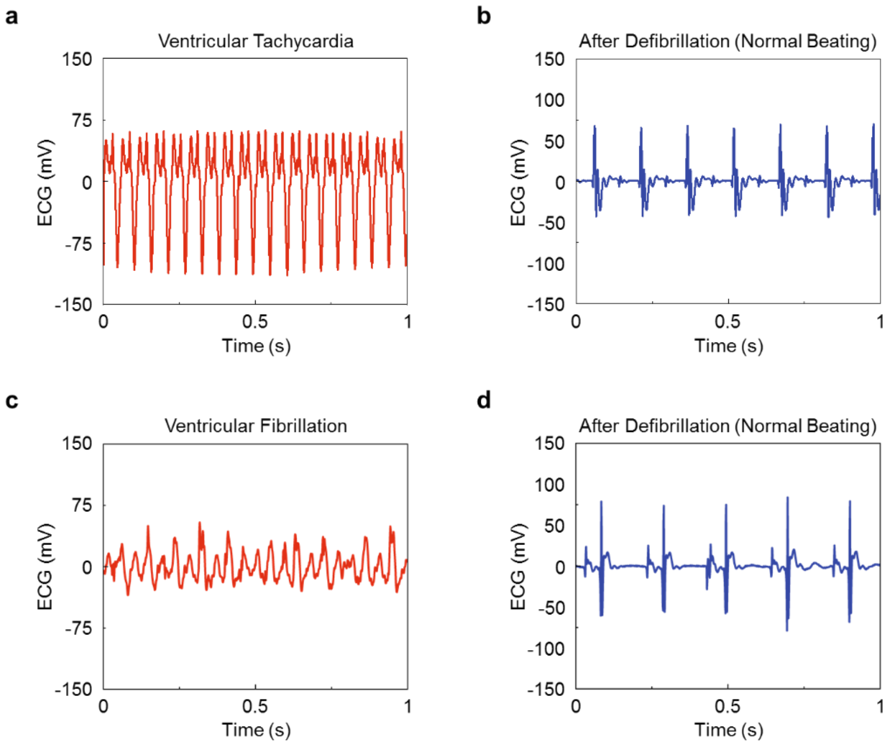

Supplementary Fig. 22 ECG traces of hearts before and after LiDB-enabled defibrillation. a and **b**, ECG traces of hearts during ventricular tachycardia (**a**) and after LiDB-enabled defibrillation (**b**). **c** and **d**, ECG traces of hearts during ventricular fibrillation (**c**) and after LiDB-enabled defibrillation (**d**).

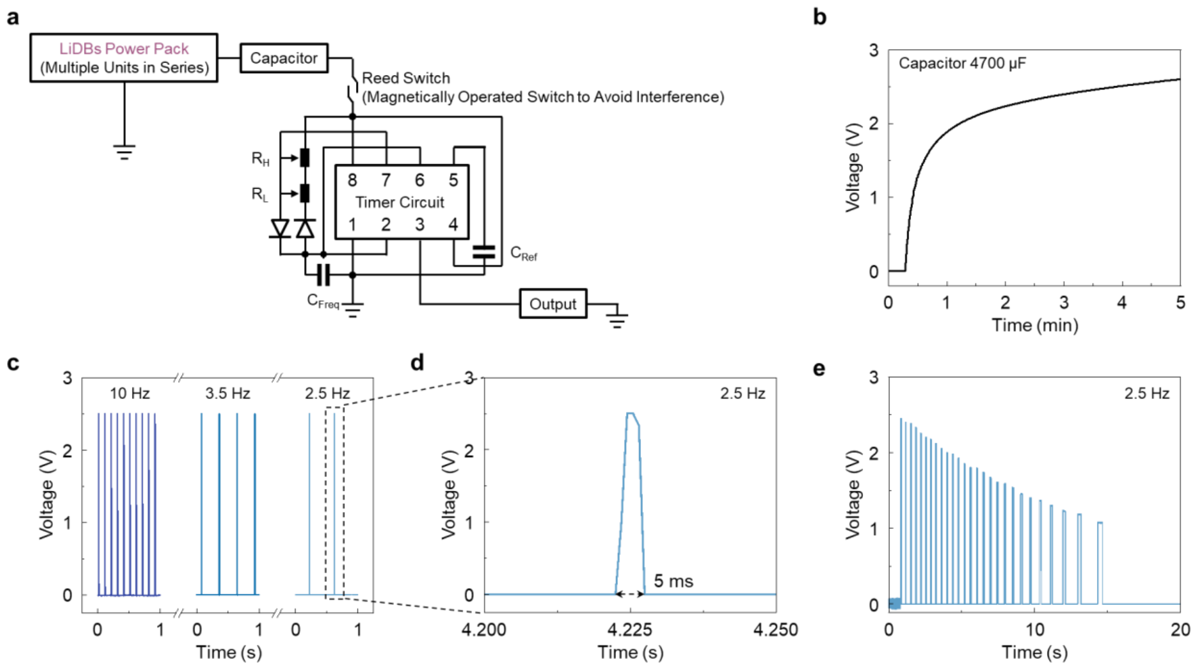

Supplementary Fig. 23 Activation of a pulse generator circuit by 6 LiDBs connected in series. a, Circuit diagram of the pulse generator circuit connected to a LiDB power pack. A reed switch was used to avoid interference. **b**, Charging curve of a 4700 μF capacitor. **c** and **d**, By tuning the value of R_H and R_L, the pulse frequency was tuned from 2.5 to 10 Hz and the pulse width was tuned to 5 ms. **e**, Output voltage of 2.5 Hz pulses in one pacing cycle. One LiDB power pack could power 3 stimulation cycles.

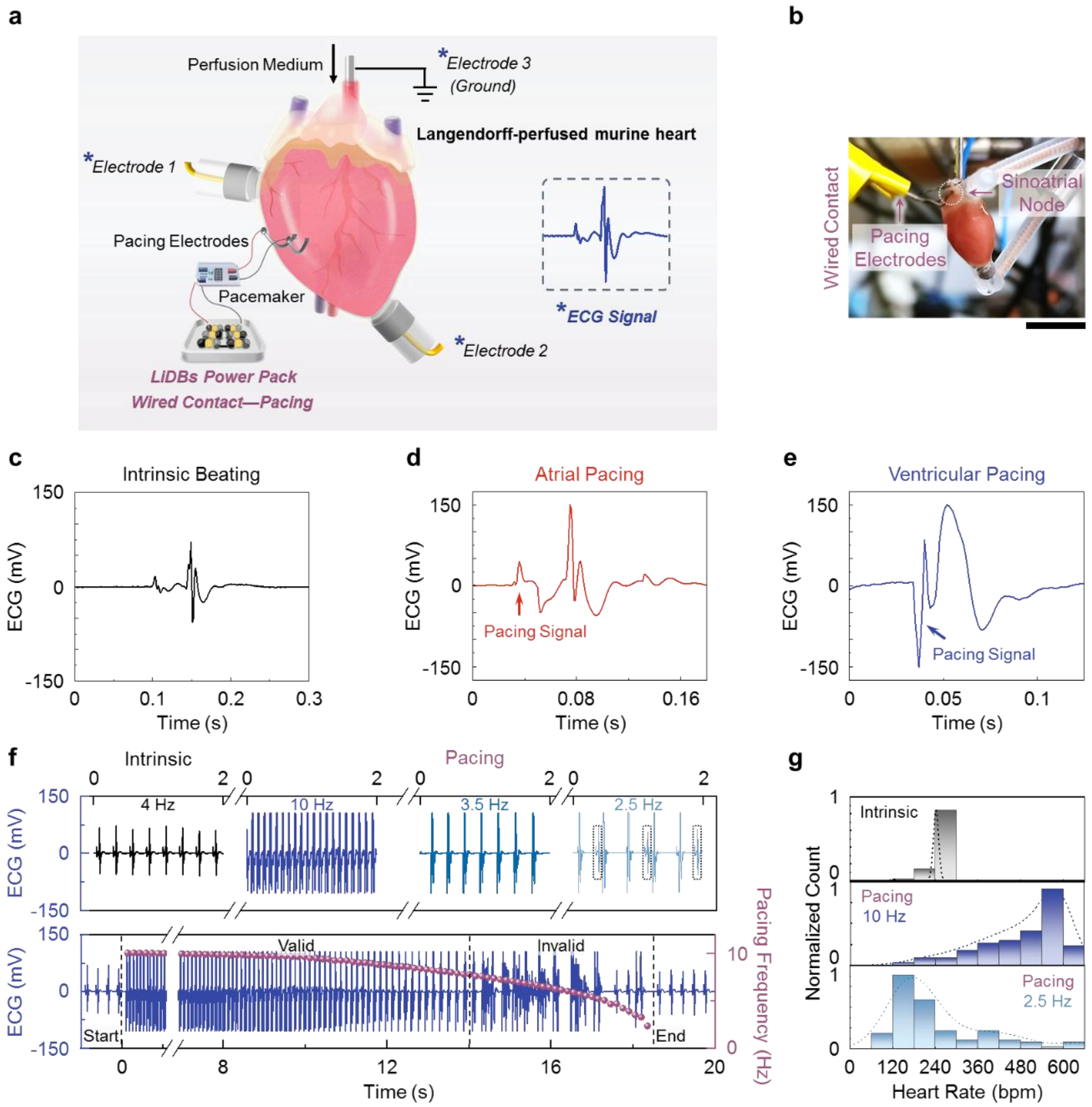

Supplementary Fig. 24 ***Ex vivo* murine heart pacing by LiDBs. a**, Wired contact for pacing. An ECG signal was monitored by three electrodes to reflect the heart responses. **b**, Image of the wired contact for atrial pacing. Scale bar, 1.2 cm. **c**–**e**, ECG spikes of an intrinsic heartbeat (**c**) and heartbeats during atrial (**d**) and ventricular (**e**) pacing powered by LiDBs. The arrow marks the time of the pacing signal. **f**, Upper, ECG signals of hearts under different pacing frequencies. Lower, ECG trace and the corresponding instantaneous pacing frequency during one 10 Hz pacing stimulation. **g**, Normalized counts of heart rates under 10 and 2.5 Hz pacing. 3 independent stimulations were counted.

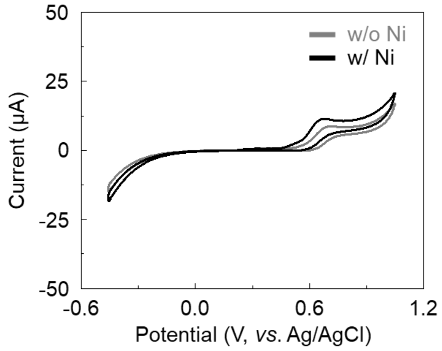

Supplementary Fig. 25 Cyclic voltammograms of the silk hydrogel without (grey) and with Ni-particles (black) at a scan rate of 10 mV s^{-1}.

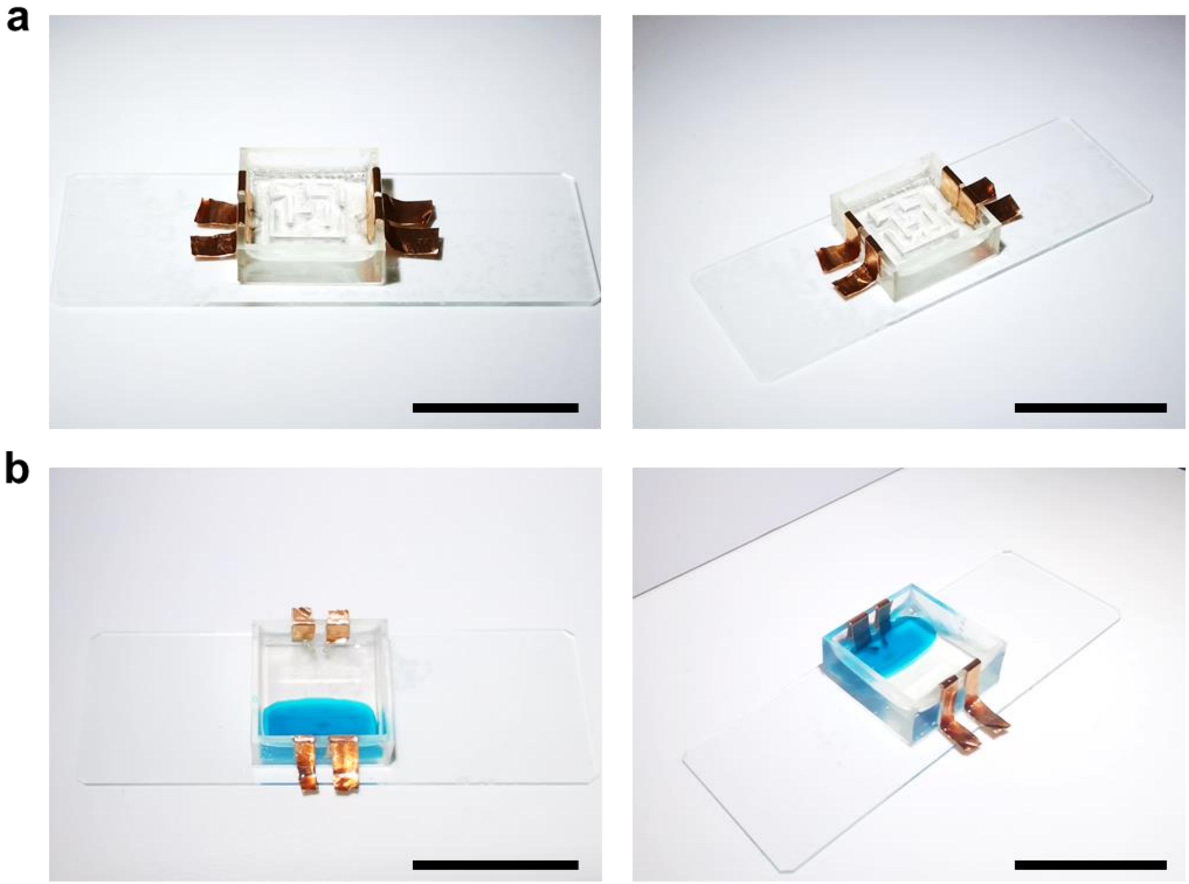

Supplementary Fig. 26 Images of the maze filled with oil (**a**) **and the double-deck well filled with oil and aqueous solution with blue dye** (**b**)**.** Scale bars, 2 cm.

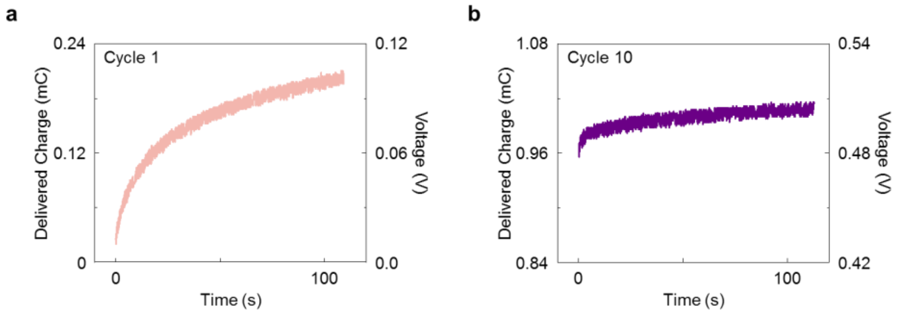

Supplementary Fig. 27 The 1st (a) and 10th (b) charging cycle of a 2 mF capacitor by the magnetically driven LiDB. The capacitor was connected to the target electrodes in the maze to collect the delivered energy of each cycle.

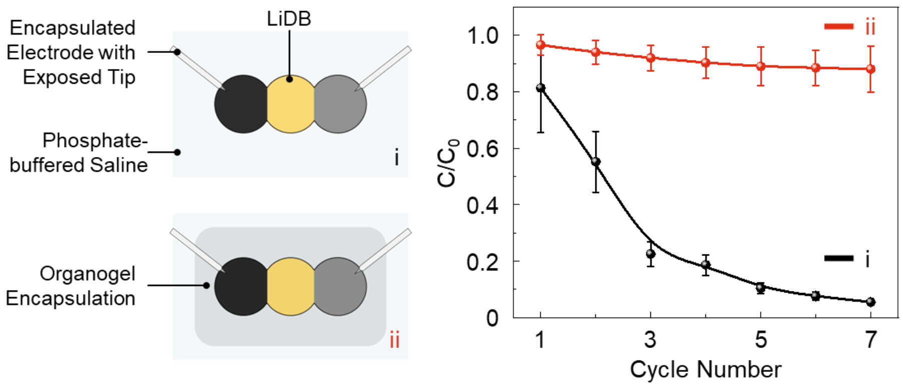

Supplementary Fig. 28 Organogel encapsulation enabled preservation of LiDBs in physiological environments. Left, Schematic showing droplets without (i) and with (ii) poly(styrene-b-ethylene-co-butylene-b-styrene) triblock copolymer encapsulation. Right, cyclic performance of LiDBs at a charging-discharging current of 1 μA. C_0 corresponds to the battery capacity before immersion in phosphate-buffered saline (pH 7.4, Gibco). C corresponds to the capacities after the charging-discharging cycle. Data are presented as mean values ± s.d. of n = 3 replicates.

Supplementary Table 1. Comparison of sizes and volumetric capacities of various hydrogel-based Li-ion batteries and LiDBs of three different volumes. Volumetric capacities of previous works[5-8] were calculated from the maximum area capacity (mAh cm^{-2}) or specific capacity (mAh g^{-1}) and the size of a battery unit.

**Hydrogel**	**Area (cm^2)**	**Thickness (cm)**	**Volumetric capacity (mAh cm^{-3})**
Poly(vinyl alcohol)[8]	8	0.95	0.067
Carboxymethyl-cellulose[9]	1	0.9	0.008
Polyacrylamide[10]	0.63	0.25	0.109
Polyacrylamide[11]	0.5	0.19	0.432
1 μL Droplets (This work)	1.21×10^{-2}	0.08	0.020
100 nL Droplets (This work)	2.60×10^{-3}	0.17	0.138
10 nL Droplets (This work)	5.61×10^{-4}	0.37	0.568

References

1. Villar, G., Graham, A. D. & Bayley, H. A tissue-like printed material. *Science* **340**, 48-52 (2013).
2. Liu, Y., Zhu, Y. & Cui, Y. Challenges and opportunities towards fast-charging battery materials. *Nat. Energy* **4**, 540-550 (2019).
3. Li, Y. *et al.* On-Chip Batteries for Dust-Sized Computers. *Adv. Energy Mater.* **12**, 2103641 (2022).
4. Zheng, Q. *et al.* In vivo powering of pacemaker by breathing-driven implanted triboelectric nanogenerator. *Adv. Mater.* **26**, 5851-5856 (2014).
5. Ouyang, H. *et al.* Symbiotic cardiac pacemaker. *Nat. Commun.* **10**, 1-10 (2019).
6. Zhang, Y. & Tao, T. H. Skin-Friendly Electronics for Acquiring Human Physiological Signatures. *Adv. Mater.* **31**, 1905767 (2019).
7. Liu, M. *et al.* Robotic Manipulation under Harsh Conditions Using Self-Healing Silk-Based Iontronics. *Adv. Sci.* **9**, 2102596 (2022).
8. Liu, Z. *et al.* Towards wearable electronic devices: A quasi-solid-state aqueous lithium-ion battery with outstanding stability, flexibility, safety and breathability. *Nano Energy* **44**, 164-173 (2018).
9. Zhao, Y. *et al.* A self-healing aqueous lithium-ion battery. *Angew. Chem. Int. Ed.* **55**, 14384-14388 (2016).
10. Wang, J. *et al.* Ultrasoft all-hydrogel aqueous lithium-ion battery with a coaxial fiber structure. *Polym. J.* **54**, 1383-1389 (2022).
11. Ye, T. *et al.* A Tissue-Like Soft All-Hydrogel Battery. *Adv. Mater.* **34**, 2105120 (2022).
